# Molecular Design, Synthesis, and Biological Evaluation of 2-Hydroxy-3-Chrysino Dithiocarbamate Derivatives

**DOI:** 10.3390/molecules24173038

**Published:** 2019-08-21

**Authors:** Pulabala Ramesh, Vankadari Srinivasa Rao, Yi-An Hong, P. Muralidhar Reddy, Anren Hu

**Affiliations:** 1 Department of Chemistry, SR&BGNR. Government College (A), Khammam 507 002, India; 2 Department of Chemistry, Osmania University, Hyderabad 500 007, India; 3 Department of Laboratory Medicine and Biotechnology, College of Medicine, Tzu-Chi University, Hualien 97071, Taiwan

**Keywords:** chrysin, epoxide, dithiocarbamates, biological activities, molecular docking studies

## Abstract

A series of 2-hydroxy-3-chrysino dithiocarbamate derivatives (**3a**–**k**) were designed, synthesized, and characterized for their structure determination by ^1^H NMR, ^13^C NMR, and HRMS (ESI) spectral data. They were screened for their in vitro biological activities against a panel of selected bacterial and fungal strains. These antimicrobial studies indicate that some of the analogues manifested significant activity compared to standard drugs. Among the synthetic analogues (**3a**–**k**), compounds **3d**, **3f**, and **3j** exhibited very good antibacterial activity and compounds **3d**, **3f**, and **3h** showed very good antifungal activity compared to the standard drugs penicillin and itrazole, respectively. The compounds **3e**, **3g**, and **3h** showed moderate antibacterial activity and the compounds **3j** and **3k** showed moderate antifungal activity. Molecular docking studies were performed and the experimental antimicrobial screening results were also correlated with the binding energy values obtained by molecular docking. The synthesized chrysin analogues (**3a**–**k**) have obeyed Lipinski’s “rule of five” and have drug-likeness.

## 1. Introduction

In the recent past, many pharmaceutical products have been designed and developed using plant based lead compounds like polyphenols [1]. Among them, flavonoids are the extensively studied biologically active compounds possessing strong antioxidant property and having potential health benefits in the prevention of cardiovascular disorders, and considered safe with a low toxicity [2,3,4,5,6]. Flavonoids also exhibit a wide variety of biological activities like antibacterial, anti-inflammatory, anti-diabetic, anti-allergic, antiviral, vasodilatory, and anticancer activities [7,8,9,10,11,12,13,14,15,16,17] and have potential to be developed or modified as effective drug candidates. Heterocyclic moieties linked to chromone system have enormous applications in pharmacological fields like antimicrobial [8,9,10,11,12,13,14,15,16,17,18,19,20,21], anti-inflammatory [22,23], anti-cancer [24,25,26,27], and anti-oxidant [28] activities.

Among the flavones class, chrysin (5,7-dihydroxy flavone) is an important biologically active compound. It is found in many medicinal plants, honey, propolis, mushrooms, and mainly isolated from an Indian medicinal plant ‘*oroxylumindicum*’ [29,30]. Chrysin is reported to exhibit various biological activities, which includes antibacterial [31], anti-inflammatory [32] anti-allergic [33], antioxidant [34], and anticancer [35] activities. Several attempts have been made to synthesize the structural derivatives of chrysin and to study their biological activities [36,37,38]. These studies indicated that synthetic analogues of chrysin are found to have more potent biological activities than standard drugs.

Epoxides are most useful intermediates for variety of synthetic reactions in organic synthesis due to ring strain. They can undergo regioselective ring opening from the less hindered terminal carbon side of the epoxide ring with a wide variety of nucleophiles by SN^2^ reaction. The dithiocarbamate nucleophiles generated in situ from CS_2_ and amines open the epoxide ring from the terminal carbon side [39,40,41] to afford 2-hydroxy dithiocarbamates, which are found to have a wide variety of applications in organic synthesis [42,43,44,45], pharmaceuticals [46,47,48,49], and agriculture [50,51,52].

In continuation to our studies of the synthetic modifications of chrysin and their biological screening [53,54], we herein report the synthesis of new 2-hydroxy-3-chrysino dithiocarbamate derivatives (**3a**–**k**). Antimicrobial studies were carried out to find the best drug candidate among the synthesized compounds (**3a**–**k**). Molecular modeling studies were also performed on these analogues (**3a**–**k**) to find the binding interaction to support the antibacterial activities. As per our knowledge, this is the first report of synthesis and antimicrobial activity studies of 2-hydroxy-3-chrysino dithiocarbamate derivatives.

## 2. Results

### 2.1. Chemistry

**Scheme 1:**
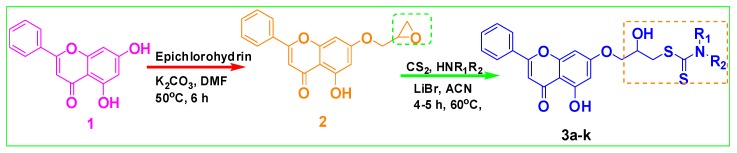
Synthesis of novel 2-hydrox-3-chrysino dithiocarbamate derivatives **3a**–**k**.

In our present study, our aim is to enhance the biological activity of chrysin by linking it to 2-hydroxy dithiocarbamates at C (7) position. This was achieved in two steps. Epoxy-methyl group was linked to chrysin at its C (7) position by reacting with epichlorohydrin in presence of K_2_CO_3_ in DMF solvent at 50 °C which gave the epoxide (2). This epoxide derivative (2) was made to react with a mixture of CS_2_ and secondary amine in acetonitrile at 60 °C to give the designed 2-hydroxy-3-chrysino dithiocarbamate derivatives (**3a**–**k**) (Scheme 1) in moderate to good yields (Table 1). The synthetic analogues (**3a**–**k**) were well characterized by ^1^H NMR, ^13^C NMR, and HRMS-ESI spectral analysis and the spectra of (**3a–k**) can be found in the supplementary materials. Formation of the designed compounds was identified by ESI-HRMS spectra with their [M + H] *m*/*z* values. The characteristic peak of the methine proton of all the synthesized compounds with chemical shift at *δ* = 4.15–4.16ppm (CHOH, ddd, *J* = 15.3, 9.5, 5.3 Hz, 1H) in ^1^H NMR spectra indicated the formation of the designed 2-hydroxy dithiocarbamate derivatives (**3a**–**k**).

### 2.2. Pharmacology

#### 2.2.1. Antimicrobial Evaluation

In order to find the potent antimicrobial agent among the synthesized compounds (**3a**–**k**), they were assessed for their in vitro antibacterial activity against *Staphylococcus epidermis* (MTCC 96) and *Bacillus subtilis* (MTCC 441) as Gram-positive, and *Escherichia coli* (MTCC 443) and *Pseudomonas aeruginosa* (MTCC 741) as Gram-negative bacteria. In vitro antifungal activities were also evaluated against *Saccharomyces cerevisiae* (MTCC 170) and *Candida albicance* (MTCC 3017) for the synthesized compounds (**3a**–**k**). To determine these preliminary antimicrobial activities, agar diffusion method [55,56] was used with penicillin and itrazole as the reference drugs to compare antibacterial and antifungal activities, respectively.

For all the synthesized compounds, the average diameter zone of inhibition round the disk in mm was recorded against the selected bacterial and fungal strains. For the selected compounds, which were showing remarkable growth in inhibition zones, minimum inhibitory concentration (MIC) in μg/mL was also measured using the two fold serial dilution method [57,58]. Most of the synthesized compounds exhibited considerable activity against the selected microorganisms and the findings of these antimicrobial studies are presented Table 2.

The compounds **3d**, **3f**, and **3j** manifested excellent antibacterial activity with zone of inhibition of >20 mm. The compounds **3e**, **3g**, and **3h** exhibited significant activity. The compounds **3a**, **3b**, **3c**, **3i**, and **3k** showed least activity with respect to the reference drug penicillin. The MIC values of **3d**, **3f**, **3g**, and **3j** also reinforce the inhibitory activity. The compounds **3d**, **3f**, and **3h** showed remarkable antifungal activity with inhibitory zone > 20 mm. The compounds **3j** and **3k** also exhibited moderate activity, which is also supported by MIC values.

#### 2.2.2. Molecular Docking Studies

In addition to the synthesis and antimicrobial screening of 2-hydroxy-3-chrysino dithiocarbamate derivatives (**3a**–**k**), molecular docking studies were also performed to elucidate the observed antimicrobial results using Molegro virtual docker (MVD-2013 (6.0)) software. These docking studies predict the drug likeness of ligands, which will give the substitutional and configurational necessities for optimum receptor pit which are essential to have best pharmacophore activity. From PDB Bank RSCB, 3D-structures of selected proteins of *E. coli* FabH (pdb id: **1HNJ**) and *S. cerevisiae* (pdb id: **5EQB**) were taken with an X-ray resolution range of 1.46 Å and 2.59 Å, respectively. First target is β-Ketoacyl-acyl-carrier protein (ACP) synthase III, also known as FabH or KAS III (pdb id: **1HNJ**), due to its important and regulatory role in bacterial fatty acid biosynthesis (FAB) [59,60], and found in both Gram-positive and Gram-negative bacteria. The enzyme FabH is found to initiate the elongation cycle of fatty acid [61,62]. It was also observed that via product inhibition, FabH is involved in the regulation of the biosynthetic pathway [63]. Some of the earlier synthesized C (7) modified chrysin derivatives were also found to inhibit FabH as antibiotics [38]. The second target is Lanosterol 14-alpha demethylase with intact transmembrane domain bound to itraconazole (*S. cerevisiae*) for showing antifungal activity. It acts as oxidoreductase inhibitor. Its organism is *S. cerevisiae* (MTCC 170). The published crystal structure of ITZ bound within the active site cavity of CYP51 (PDB ID:**5EQB**) served as a useful template for generating proposed binding modes with respect to antifungal activity.

Investigations were carried out to evaluate the interaction between the ligands and the receptor, their fitness function ability of transferase, oxido-reductase proteins with different inhibitors. The active site pocket of these proteins consist of Arg36, Trp32, Thr28, Arg151, Ser29, Asp27, Ile55, Gly152, Gly209, Asn210, Lys214, Phe213, Ala208, Met207, Arg249, Thr37, Arg56, and Ile156 amino acid residues. In the active site regions of 1HNJ protein, Thr81, Gly306, Phe304, Gly305, Asn210, Arg249, Ala246, Leu142, Cys112, Val212, Met207, Lys214, Gly306, and Leu189 amino acid residues can play important roles. In 5EQB protein, Met25, Ser61, Ile62, Pro63, Leu69, and Lys24 amino acid residues can play important roles.

**Figure 1:**
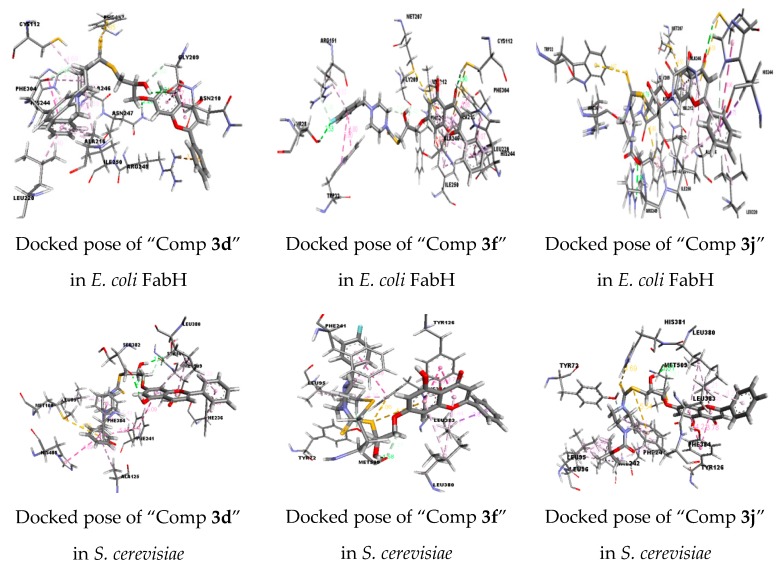
(**3d**, **3f**, and **3j**) docked into the binding site of *E. coli* FabH (**1HNJ**) enzyme and *S. cerevisiae* (**5EQB**) with Discovery studio client 4.2.

Three-dimensional conformations of the synthesized 2-hydroxy-3-chrysino dithiocarbamate derivatives (**3a**–**k**) were generated. These structures were then docked into the active site of protein structures of *E. coli* FabH (**1HNJ**) and *S. cerevisiae* (**5EQB**) using the Molegro virtual docking software package. This will give the binding interaction of the ligand with the proteins and understanding of the possible mechanism of action. By knowing the putative binding site and docked poses with Cys–His–Asn residues of *E. coli* FabH (**1HNJ**) and *S. cerevisiae* (**5EQB**), enzymes were generated based on their binding energy with manual inspection. The synthesized analogues (**3a**–**k**) with the binding site interactions of the synthesized analogues (**3a**–**k**) with *E. coli* FabH (**1HNJ**) and *S. cerevisiae* (**5EQB**) enzymes are shown in Figure 1.

The molecules having optimum lipophilicity, maximum H-bonding ability with minimum clashes are needed to dock for good fit in the active site region of the target receptor. For the docked compounds, binding affinity values were found to be in terms of negative binding energy kcal mol^−1^. The ligands with relatively more negative binding energy will be more potent in binding with the protein. Docking data for all the synthesized compounds (**3a**–**k**) were generated and presented in Table 3 and Table 4. The docking results showed that the synthetic analogues (**3a**–**k**) were bound in the active sites of the enzymes by forming combination of hydrogen bonds, hydrophobic, and van der Waals interactions. The molecular docking results showed good binding score values for all the synthesized molecules compared to standard drugs.

Based on the Moldockscore [Grid] (kcal/mol), it is clear that from the synthesized 2-hydroxy-3-chrysino dithiocarbamate derivatives (**3a**–**k**)**,** the compounds **3d–h** and **3j** showed greater binding affinity with the protein of *E. coli* of FabH (**1HNJ**) compared to standard drug penicillin. All the synthesized compounds (**3a**–**k**) showed lower binding affinity with the protein *S. cerevisiae* (**5EQB**) compared to standard drug itrazole.

#### 2.2.3. Adsorption, Distribution, Metabolism, and Excretion (ADME)-Profile

The properties like adsorption, distribution, metabolism, and excretion (ADME) are important for any compound to be developed as a successful drug. In our present study, the Molinspiration online property calculation toolkit [64] was used to determine the properties like molecular volume (MV), molecular weight (MW), logarithm of partition coefficient (m_i_LogP), number of hydrogen bond acceptors (n-ON), number of rotatable bonds (n-ROTB), and Lipinski’s rule of five [65]. Absorption (% ABS) was calculated by: %ABS = 109 − [0.345 × Total Polar Surface Area (TPSA)] [66]. Drug-likeness model score is computed by using the Molsoft software [67], which is a collective property of physico-chemical properties, pharmacokinetics and pharmacodynamics. The pharmacokinetic parameters were calculated for the known inhibitors, synthesized analogues (**3a**–**k**), and the standard drugs, shown in Table 5.

For any molecule likely to be developed as an orally active drug candidate, it should not exhibit more than one violation from the following four criteria: m_i_LogP (octanol-water partition coefficient) ≤ 5, molecular weight ≤ 500, number of hydrogen bond acceptors ≤ 10, and number of hydrogen bonds ≤ 5. It is observed that the synthesized 2-hydroxy dithiocarbamates linked chrysin derivates (**3a**–**k**) exhibited good % absorption (% ABS) ranging from 70.12% to 80.31%. All the synthesized compounds, except **3d**, **3f**, and **3h** of **3a**–**k** obeyed Lipinski’s rule of five (number of hydrogen bond acceptors (n-ON) ≤ 10) and obeyed the requirement to be an orally active drug candidate. Hence, the synthesized derivatives (**3a**–**k**) have good potential for subsequent development in drug discovery.

## 3. Materials and Methods

### 3.1. Chemistry

#### 3.1.1. General

Melting points of the synthesized compounds were determined in open capillaries and are uncorrected. ^1^H NMR spectra were recorded on Bruker-500 (500 MHz) spectrometer (Bruker, Fallanden, Switzerland), using deutero-chloroform (CDCl_3_) as solvent and tetramethylsilane (TMS) as an internal standard. ^13^C NMR spectra were obtained with Bruker-500 (125 MHz) spectrometer by using CDCl_3_ + DMSO-*d6* as solvent. Chemical shifts are given in parts per million (δ) and coupling constants (*J*) in Hz. Mass spectra were recorded on LC-QTOF MS mass spectrometer and given in mass units (*m*/*z*). Thin layer chromatography (TLC) was performed on Merck silica gel 60 F_254_ precoated aluminum sheets.

#### 3.1.2. Synthesis of 5-hydroxy-7-(oxiran-2-ylmethoxy)-2-phenyl-4H-chromen-4-one (**2**):

To a solution of chrysin **1** (4 mmol) in DMF (12 mL), was added K_2_CO_3_ (20 mmol) and the mixture was stirred for 20 min at room temperature. Epichlorohydrin (20 mmol) was then added drop wise to above mixture. The reaction mixture was heated at 50 °C for 6 h. After completion of reaction, the mixture was poured into the ice water. The precipitate and the extractions were combined and subjected to column chromatography (silica gel; eluent: PE:EA = 10:1) to afford **2** as pale yellow solid (780 mg, 64%).

m.p: 165–167 °C; ^1^H NMR (500MHz, CDCl_3_) *δ* 12.73 (s, 1H), 7.89–7.86 (m, 2H), 7.53 (dd, *J* = 17.2, 6.6 Hz, 3H), 6.67 (s, 1H), 6.53 (d, *J* = 2.2 Hz, 1H), 6.38 (d, *J* = 2.2 Hz, 1H0, 4.34 (dd, *J* = 11.0, 2.9 Hz, 1H), 4.01 (dd, *J* = 11.0, 5.9 Hz, 1H), 3.39 (dt, *J* = 6.8, 2.8 Hz, 1H), 2.98–2.93 (m, 1H), 2.79 (dd, *J* = 4.8, 2.6 Hz, 1H0; ^13^C NMR (125 MHz, CDCl_3_) *δ* 182.4, 164.3, 164.07, 162.2, 157.72, 131.8, 131.2, 129.8, 126.3, 106.0, 105.9, 98.6, 93.3, 69.2, 49.2, 44.5; HRMS (ESI): *m*/*z* calcd. for C_18_H_14_O_5_ [M + H]^+^ 311.0919, found 311.0920.

#### 3.1.3. Typical Procedure for the Synthesis of ***3*a**–**k**

To a solution of secondary amine (1.5 mmol) in ACN (10 mL), CS_2_ (3 mmol) was added drop wise. The reactions mixture was then stirred at room temperature for 30 min. To this reaction mixture, **2** (1 mmol) and LiBr (0.04 mmol) were added and then stirred at 60 °C for an appropriate time as monitored by TLC. After completion, the reaction mixture was diluted with ice cold water and extracted with EtOAc. Then, evaporation of EtOAc gave a crude residue which was further purified by column chromatography (silica gel, ethyl acetate/hexane as eluent) to afford the designed products (**3a**–**k**).

*2-Hydroxy-3-((5-hydroxy-4-oxo-2-phenyl-4H-chromen-7-yl)oxy) propyl piperidine-1-carbodithioate* (**3a**): m.p: 134–136 °C; ^1^H NMR (500 MHz, CDCl_3_) *δ* 12.71 (s, 1H), 7.89 (dd, *J* = 8.1, 1.5 Hz, 2H), 7.59–7.49 (m, 3H), 6.67 (s, 1H), 6.56 (d, *J* = 2.2 Hz, 1H), 6.41 (d, *J* = 2.2 Hz, 1H), 4.40–4.35 (m, 1H), 4.31 (s, 2H), 4.15 (ddd, *J* = 15.3, 9.5, 5.4 Hz, 1H), 3.95 (s, 2H), 3.85 (dd, *J* = 14.6, 4.3 Hz, 2H), 3.68 (dd, *J* = 14.6, 6.8 Hz, 2H), 3.28 (d, *J* = 4.4 Hz, 1H), 1.73 (br.s, 6H); ^13^C NMR (125 MHz, CDCl_3_) *δ* 195.5, 182.4, 164.4, 164.1, 162.2, 157.7, 131.8, 131.3, 129.1, 126.3, 106, 105.9, 98.8, 93.2, 71, 69.5, 39.5, 24.2; HRMS (ESI): *m*/*z* calcd. for C_24_H_25_NO_5_S_2_ [M + H]^+^ 472.1252, found 472.1258.

*2-Hydroxy-3-((5-hydroxy-4-oxo-2-phenyl-4H-chromen-7-yl)oxy)propyl pyrrolidine-1-carbodithioate* (**3b**): m.p: 144–146 °C; ^1^H NMR (500 MHz, CDCl_3_) *δ* 12.71 (s,1H), 7.89 (dd, *J* = 8.1, 1.5 Hz, 2H), 7.59–7.49 (m, 3H), 6.67 (s, 1H), 6.56 (d, *J* = 2.2 Hz, 1H), 6.41 (d, *J* = 2.2 Hz, 1H), 4.40–4.35 (m, 1H), 4.31 (s, 2H), 4.15 (ddd, *J* = 15.3, 9.5, 5.4 Hz, 1H), 3.95 (s, 2H), 3.85 (dd, *J* = 14.6, 4.3 Hz, 2H), 3.68 (dd, *J* = 14.6, 6.8 Hz, 2H), 3.28 (d, *J* = 4.4 Hz, 1H), 1.73 (br.s, 6H); ^13^C NMR (125 MHz, CDCl_3_) *δ* 195.5, 182.4, 164.4, 164.1, 162.2, 157.7, 131.8, 131.3, 129.1, 126.3, 106, 105.9, 98.8, 93.2, 71, 69.5, 39.5, 24.2; HRMS (ESI): *m*/*z* calcd. for C_23_H_23_NO_5_S_2_ [M + H]^+^ 458.1096, found 458.1106.

*2-Hydroxy-3-((5-hydroxy-4-oxo-2-phenyl-4H-chromen-7-yl)oxy)propyl morpholine-4-carbodithioate* (**3c**): m.p: 146–150 °C; ^1^H NMR (500 MHz, CDCl_3_) *δ* 12.71 (s, 1H), 7.89 (dd, *J* = 8.1, 1.5 Hz, 2H), 7.57–7.50 (m, 3H), 6.68 (s, 1H), 6.55 (d, *J* = 2.2 Hz, 1H), 6.40 (d, *J* = 2.2 Hz, 1H), 4.42–4.35 (m, 2H), 4.15 (ddd, *J* = 15.4, 9.6, 5.3 Hz, 1H), 4.02 (br.s., 2H), 3.85 (dd, *J* = 14.5, 4.3 Hz, 1H), 3.79 (s, 4H), 3.69 (dd, *J* = 14.5, 7.0 Hz, 1H); ^13^C NMR (125 MHz, CDCl_3_) *δ* 197.5, 182.5, 164.3, 164.1, 162.2, 157.7, 131.9, 131.3, 129.1, 126.3, 106.0, 105.9, 98.7, 93.2, 71.0, 69.3, 39.4. HRMS (ESI): *m*/*z* calcd. for C_23_H_23_NO_6_S_2_ [M + H]^+^ 474.1045, found 474.1043.

*2-Hydroxy-3-((5-hydroxy-4-oxo-2-phenyl-4H-chromen-7-yl)oxy)propyl 4-benzylpiperazine-1-carbodithiote* (**3d**): m.p: 154–156 °C; ^1^H NMR (500 MHz, CDCl_3_) *δ* 12.71 (s, 1H), 7.89 (dd, *J* = 8.1, 1.5 Hz, 2H), 7.59–7.49 (m, 3H), 7.37–7.30 (m, 5H), 6.68 (s, 1H), 6.55 (d, *J* = 2.2 Hz, 1H), 6.40 (d, *J* = 2.2 Hz, 1H), 4.37 (s, 3H), 4.15 (ddd, *J* = 15.4, 9.5, 5.3 Hz, 1H), 3.99 (s, 2H), 3.84 (dd, *J* = 14.6, 4.3 Hz, 1H), 3.67 (dd, *J* = 14.6, 6.9 Hz, 1H), 3.55 (s, 2H), 3.18 (s, 1H), 2.56 (br.s., 4H); ^13^C NMR (125 MHz, CDCl_3_) *δ* 196.6, 182.4, 164.4, 164.1, 162.2, 157.7, 137.2, 131.8, 131.3, 129.1, 128.4, 127.4, 126.3, 106, 105.9, 98.8, 93.2, 71, 69.4, 62.4, 52.3, 39.5; HRMS (ESI): *m*/*z* calcd. for C_30_H_30_N_2_O_5_S_2_ [M + H]^+^ 563.1674, found 563.1686.

*2-Hydroxy-3-((5-hydroxy-4-oxo-2-phenyl-4H-chromen-7-yl)oxy)propyl thiomorpholine-4-carbodithioate* (**3e**): m.p: 168–170 °C; ^1^H NMR (500 MHz, CDCl_3_) *δ* 12.72 (s, 1H), 7.89 (dd, *J* = 8.1, 1.5 Hz, 2H), 7.58–7.50 (m, 3H), 6.68 (s, 1H), 6.56 (d, *J* = 2.2 Hz, 1H), 6.40 (d, *J* = 2.2 Hz, 1H), 4.64 (br.s, 2H), 4.41–4.34 (m, 2H), 4.32 (br.s, 1H), 4.15 (ddd, *J* = 15.4, 9.5, 5.3 Hz, 1H), 3.85 (dd, *J* = 14.5, 4.3 Hz, 1H), 3.68 (dd, *J* = 14.5, 6.9 Hz, 1H), 3.07 (d, *J* = 4.5 Hz, 1H), 2.78 (s, 4H); ^13^C NMR (125 MHz, CDCl_3_+DMSO-*d*_6_) *δ* 196.7, 182.4, 164.5, 164.1, 162.1, 157.7, 131.8, 131.2, 129.1, 126.3, 105.9, 105.8, 98.8, 93.2, 71.2, 68.9, 29.6, 27.2; HRMS (ESI): *m*/*z* calcd. for C_23_H_23_NO_5_S_3_ [M + H]^+^ 490.0817, found 490.0822.

*2-Hydroxy-3-((5-hydroxy-4-oxo-2-phenyl-4H-chromen-7-yl)oxy)propyl 4-(4-fluorophenyl) piperazine-1-carbodithiate* (**3f**): m.p: 146–148 °C; ^1^H NMR (500 MHz, CDCl_3_) *δ* 12.72 (s, 1H), 7.89 (d, *J* = 8.3 Hz, 2H), 7.54 (m, 3H), 7.03–6.95 (m, 2H), 6.88 (dd, *J* = 9.1, 4.5 Hz, 2H), 6.68 (s, 1H), 6.56 (d, *J* = 2.1 Hz, 1H), 6.41 (d, *J* = 2.2 Hz, 1H), 4.52 (br.s, 2H), 4.45–4.36 (m, 1H), 4.16 (ddd, *J* = 15.3, 9.5, 5.3 Hz, 1H), 3.86 (dd, *J* = 14.6, 4.3 Hz, 1H), 3.70 (dd, *J* = 14.6, 4.3 Hz, 1H), 3.26–3.18 (m, 4H), 3.13 (d, *J* = 4.5 Hz, 1H); ^13^C NMR (125 MHz, CDCl_3_) *δ* 197.2, 182.4, 164.3, 164.1, 162.2, 158.7, 157.7, 156.8, 146.88 (d, *J* = 2.4 Hz), 131.8, 131.3, 129.1, 126.3, 118.46 (d, *J* = 7.8 Hz), 115.9, 115.7, 106, 105.9, 98.7, 93.2, 71, 69.3, 49.9, 39.3; HRMS (ESI): *m*/*z* calcd. for C_29_H_27_FN_2_O_5_S_2_ [M + H]^+^ 567.1424, found 567.1439.

*2-Hydroxy-3-((5-hydroxy-4-oxo-2-phenyl-4H-chromen-7-yl)oxy)propyl 4-(pyridine-2-yl) piperazine-1-carbodithioate* (**3g**): m.p: 152–154 °C; ^1^H NMR (500 MHz, CDCl_3_) *δ* 12.71 (s, 1H), 8.20 (dd, *J* = 4.9, 1.2 Hz, 1H), 7.89 (dd, *J* = 8.0, 1.5 Hz, 2H), 7.59–7.48 (m, 4H), 6.71–6.69 (m, 1H), 6.68 (d, *J* = 2.5 Hz, 1H), 6.63 (d, *J* = 1H), 6.56 (d, *J* = 2.1 Hz, 1H), 6.41 (d, *J* = 2.2 Hz, 1H), 4.49 (s, 1H), 4.40 (s, 1H), 4.18 (td, *J* = 11.9, 6.0 Hz, 2H), 4.15 (ddd, *J* = 15.3, 9.5, 5.4 Hz, 1H), 3.86 (dt, *J* = 18.6, 9.3 Hz, 1H), 3.70 (m, 6H), 3.19 (s, 1H); ^13^C NMR (125 MHz, CDCl_3_ + DMSO-*d*_6_) *δ* 196.6, 182.3, 164.9, 164, 161.9, 158.4, 157.7, 147.8, 137.7, 131.9, 131.1, 129.1, 126.3, 113.8, 107, 105.7, 105.6, 98.9, 93.3, 71.8, 68.1, 44.2; HRMS (ESI): *m*/*z* calcd. for C_28_H_27_N_3_O_5_S_2_ [M + H]^+^ 550.1470, found 550.1492.

*2-Hydroxy-3-((5-hydroxy-4-oxo-2-phenyl-4H-chromen-7-yl)oxy)propyl 4-(4-methoxyphenyl) piperazine-1-carbodithioate* (**3h**): m.p: 151–153 °C; ^1^H NMR (500 MHz, CDCl_3_) *δ* 12.71 (s, 1H), 7.88 (d, *J* = 8.3 Hz, 2H), 7.59–7.47 (m, 3H), 6.90 (d, *J* = 9.1 Hz, 2H), 6.85 (d, *J* = 9.1 Hz, 2H), 6.67 (s, 1H), 6.56 (d, *J* = 2.1 Hz, 1H), 6.41 (d, *J* = 2.2 Hz, 1H), 4.51 (br. s, 2H), 4.39 (dd, *J* = 10.2, 4.6 Hz, 1H), 4.16 (ddd, *J* = 15.3, 9.5, 5.3 Hz, 1H), 3.86 (dd, *J* = 14.6, 4.3 Hz, 1H), 3.77 (s, 3H), 3.70 (dd, *J* = 14.6, 6.9 Hz, 1H), 3.21–3.15 (m, 5H); ^13^C NMR (125 MHz, CDCl_3_) δ 197, 182.4, 164.4, 164.1, 162.2, 157.7, 154.6, 144.5, 131.8, 131.3, 129.1, 126.3, 118.9, 114.6, 106, 105.9, 98.8, 93.2, 71, 69.4, 55.6, 50.5, 39.6; HRMS (ESI): *m*/*z* calcd. for C_30_H_30_N_2_O_6_S_2_ [M + H]^+^ 579.1624, found 579.1627.

*2-Hydroxy-3-((5-hydroxy-4-oxo-2-phenyl-4H-chromen-7yl)oxy)propyl cis-3,5-dimethylmorpholine-4-carbodithioate* (**3i**): m.p: 181–183 °C; ^1^H NMR (500 MHz, CDCl_3_) *δ* 12.72 (s, 1H), 7.89 (dd, *J* = 8.1, 1.5 Hz, 2H), 7.63–7.43 (m, 3H), 6.68 (s, 1H), 6.55 (d, *J* = 2.2 Hz, 1H), 6.40 (d, *J* = 2.2 Hz, 1H), 5.45 (s, 1H), 4.51 (s, 1H), 4.44–4.33 (m, 1H), 4.15 (ddd, *J* = 15.4, 9.5, 5.3 Hz, 1H), 3.85 (dd, *J* = 14.5, 4.3 Hz, 1H), 3.68 (dd, *J* = 14.5, 6.9 Hz, 3H), 3.13 (d, *J* = 4.4 Hz, 1H), 2.95 (s, 1H), 2.79 (s, 1H), 1.26 (s, 3H), 1.25 (s, 3H); ^13^C NMR (125 MHz, CDCl_3_) *δ* 196.9, 182.4, 164.3, 164.1, 162.3, 157.7, 131.8, 131.3, 129.1, 126.3, 106, 105.9, 98.7, 93.2, 71, 69.3, 39.3, 18.5; HRMS (ESI): *m*/*z* calcd. for C_25_H_27_NO_6_S_2_ [M + H]^+^ 502.1358, found 502.1366.

*tert-Butyl 4-(((2-hydroxy-3-((5-hydroxy-4-oxo-2-phenyl-4H-chromen-7-yl)oxy)propyl)thio) carbonothioyl)piperazine-1-carboxylate* (**3j**): m.p: 140–142 °C; ^1^H NMR (500 MHz, CDCl_3_) δ 12.72 (s, 1H), 7.89 (d, *J* = 6.6 Hz, 2H), 7.61–7.48 (m, 3H), 6.68 (s, 1H), 6.56 (d, *J* = 2.2 Hz, 1H), 6.40 (d, *J* = 2.2 Hz, 1H), 4.38 (dd, *J* = 10.5, 5.7 Hz, 2H), 4.15 (ddd, *J* = 15.4, 9.5, 5.3 Hz, 1H), 4.00 (br.s, 2H), 3.85 (dd, *J* = 14.6, 4.3 Hz, 1H), 3.68 (dd, *J* = 14.6, 7.0 Hz, 1H), 3.60–3.54 (m, 4H), 3.11 (d, *J* = 4.4 Hz, 1H), 1.48 (s, 9H); ^13^C NMR (125 MHz, CDCl_3_) δ 197.5, 182.4, 164.3, 164.1, 162.2, 157.7, 154.4, 131.6, 131.3, 129.1, 126.3, 106, 105.9, 98.7, 93.2, 80.7, 71, 69.3, 39.6, 28.3; HRMS (ESI): m/z calcd. for C_28_H_32_N_2_O_7_S_2_ [M + H]^+^ 573.1729, found 573.1731.

*2-Hydroxy-3-((5-hydroxy-4-oxo-2-phenyl-4H-chromen-7-yl)oxy)propyl diethyl carbamodithioate* (**3k**): m.p: 158–160 °C; ^1^H NMR (500 MHz, CDCl_3_) *δ* 12.71 (s, 1H), 7.89 (d, *J* = 6.7 Hz, 2H), 7.60–7.47 (m, 3H), 6.68 (s, 1H), 6.56 (d, *J* = 2.2 Hz, 1H), 6.40 (d, *J* = 2.2 Hz, 1H), 4.38 (dd, *J* = 11.2, 5.2 Hz, 1H), 4.15 (ddd, *J* = 15.3, 9.5, 5.4 Hz, 1H), 4.05 (dt, *J* = 10.8, 6.7 Hz, 2H), 3.82 (m, 3H), 3.67 (dd, *J* = 14.7, 6.8 Hz, 1H), 3.35 (d, *J* = 4.4 Hz, 1H), 1.33 (t, *J* = 7.1 Hz, 3H), 1.29 (t, *J* = 7.1 Hz, 3H); ^13^C NMR (125 MHz, CDCl_3_) *δ* 195.7, 182.4, 164.4, 164, 162.2, 157.7, 131.8, 131.3, 129, 126.3, 106, 105.9, 98.8, 93.2, 71, 69.5, 50.2, 47.1, 39.5, 12.5, 11.5; HRMS (ESI): *m*/*z* calcd. for C_23_H_25_NO_5_S_2_ [M + H]^+^ 460.1252, found 460.1256.

### 3.2. Preparation of the Protein and the Ligand for Docking Simulations

Chemdrawsuite [68] was used to generate the optimized structures of the synthesized analogues (**3a**–**k**) and energy was minimized using OPLS 2005 force field [69] through Ligprep module of Schrodinger Suite 2013 [70]. Docking simulations were carried on the optimized structures of the synthesized compounds using .sdf format.

The crystal structure of *E. coli* FabH with accession number (1HNJ) was retrieved from the Protein Data Bank (PDB) for docking simulations. Molegro Virtual Docker, version 5.5, was used to perform the docking. Protein structure for the docking procedure was prepared by removing the solvent molecules and structural parameters of ligands like hybridization, bond order, precise hydrogen atoms were assigned using Molegro Virtual Docker software. Based on the requirement, charges were assigned. Detect cavities option was used to obtain possible binding sites in preparation tools and five cavities were obtained. The cavity around the anion binding site (volume of 177 Å^3^) was used for docking calculations and further modified using side chain minimization. Grid-based Mol-Dock score (GRID) function was used to carry docking calculations with a grid resolution of 0.20 Å. Based on the Mol Dock score and Rerank score, the best ligand poses were chosen. The docking calculations were performed with a dual processor, Windows 7 based computer with 4 GB RAM and each docking process took 10–15 min. COOT graphical program [71] was used to perform molecular alignment with ALIGN program. Protein-ligand interaction studies were performed using Accelrys Discovery Studio v3.5 [72].

### 3.3. Docking Simulation of the Synthesized Compounds

Molgro Virtual Docker 2010.4.0 molecular docking program predicts the interaction of the molecules with a protein receptor. The structure based virtual screening of the compounds was carried based on Mol Dock scoring function (MolDock Score) derived from the Piecewise Linear Potential (PLP) scoring functions [73]. Further, the total energy was minimized using Melder Mead Simplex Minimization using non-grid force field and H-bond directionality [74]. Protein–receptor interactions with the compound were evaluated based on internal electrostatic, hydrogen bond interactions and sp^2^–sp^2^ torsions and binding affinity. The synthesized compounds with highest binding affinity against *E. coli* FabH protein (**1HNJ**), *S. cerevisiae* (**5EQB**) were selected as a function of Moldock score.

### 3.4. Biological Activity and ADME Properties of Compounds

For all the synthesized compounds, drug-likeness was evaluated using Lipinski filters and biological activity was predicted using Molinspiration webserver (©Molinspiration Cheminformatics 2018).

### 3.5. Softwares, Suites, and Webservers

MarvinSketch 5.6.0.2 (1998–2011, Copyright © Chem Axon Ltd.) was used to design the compounds and they were docked using Molegro Virtual Docker 2010.4.0.0. For the molecular visualizations, Accelrys Discovery Studio^®^ Visualiser 3.5.0.12158 (Copyright © 2005-12, Accelrys Software Inc.) was used and various solubility parameters were calculated by applying QikProp module of Schrodinger suite 2013.

Computer-aided drug design was used for developing potential *E. coli* FabH protein (**1HNJ**), *S. cerevisiae* (**5EQB**) organism inhibitors, which enable the prediction of the ligand-binding site and to suggest possible interactions with the ligands. Molecular docking simulations were performed based on the binding model for the synthesized analogues (**3a**–**k**) with the proteins of *E. coli* FabH and *S. cerevisiae*. Putative interactions proposed by the best docked position were used as a template to evaluate the drug candidates. The active site of *E. coli* FabH generally contains Cys–His–Asn catalytic triad tunnel which is sustained in various bacteria and is important in the regulation of chain elongation and substrate binding. The interaction between Cys and substrate plays a key role in substrate binding, since the alkyl chain of CoA is broken by Cys of the catalytic triad of *E. coli* FabH.

## 4. Conclusions

In conclusion, a series of novel 2-hydroxy-3-chrysino dithiocarbamate analogues (**3a**–**k**) were synthesized in moderate to good yields and assessed for their in vitro antimicrobial activities. These antimicrobial studies indicated that most of the derivatives manifested moderate to good biological activities compared to the standard drugs penicillin and itrazole. Among the synthesized analogues, **3d**, **3f**, and **3j** showed remarkable antimicrobial activities. In addition to the antimicrobial screening, molecular modeling studies were also performed to support these biological activities, providing further insight into the interactions of the synthesized ligands with the protein of *E. coli* FabH and *S. cerevisiae*. These docking scores are in good correlation with the experimental antimicrobial results. We hope these studies will be useful in developing the new drug entities as potential chemotherapeutic agents in controlling the microbial epidemics.

## Figures and Tables

**Table 1:** Synthesis of novel 2-hydroxy-3-chrysino dithiocarbamate derivatives **3a**–**k** catalyzed by LiBr ^a^.

S.No	Entry	HNR_1_R_2_	Product ^b^	Yield ^c^ (%)	M.P (°C)
1	**3a**	Piperazine	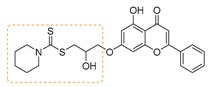	89	134–136
2	**3b**	Pyrrolidine	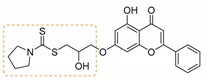	88	144–146
3	**3c**	Morpholine	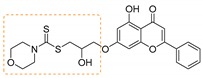	88	146–148
4	**3d**	4-Benzyl piperazine	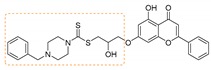	81	154–156
5	**3e**	Thiomorpholine	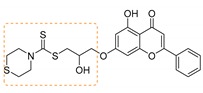	82	168–170
6	**3f**	4-Fluorophenyl piperazine	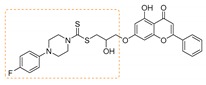	74	146–148
7	**3g**	4-Pyridyl piperazine	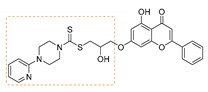	68	152–154
8	**3h**	4-Methoxyphenyl piperazine	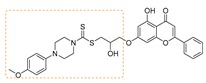	73	151–152
9	**3i**	Cis-3,5-Dimethyl morpholine	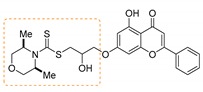	71	181–183
10	**3j**	4-Benzyloxy carbonyl piperazine	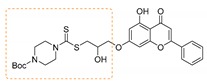	66	140–142
11	**3k**	Diethyl amine	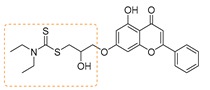	85	158–160

^a^ All the reactions were performed with CS_2_ (3 equiv), cyclic/secondary amines (1.5 equiv), and chrysin (1 equiv) catalyzed by LiBr (40 mol%). ^b^ All the products were characterized by ^1^H NMR, ^13^C NMR, and HRMS-ESI spectroscopies. ^c^ Yields refer to isolated products (based on Epoxide **2**).

**Table 2:** Antimicrobial screening of the synthesized 2-hydroxy-3-hydroxy dithiocarbamate derivatives (**3a**–**k**).

Zone of Inhibition *^a^* (mm) and MIC *^b^* (μg/mL) Values of Compounds						
	Gram-Positive Bacteria		Gram-Negative Bacteria		Fungi	
Compound Code	*S. epidermidis*	*B. subtilis*	*E. coli*	*P. aeruginosa*	*S. cerevisiae*	*C. albicans*
**3a**	16	14	17	10	8	6
**3b**	14	10	11	13	7	8
**3c**	10	8	9	11	9	6
**3d**	26 (9.37)	30 (4.68)	29 (4.68)	23 (18.75)	23 (18.75)	21 (18.75)
**3e**	18	19	22	19	8	9
**3f**	23 (18.75)	26 (9.37)	25 (9.37)	21 (18.75)	27 (9.37)	21 (18.75)
**3g**	21 (18.75)	18 (18.75)	19 (9.37)	20 (18.75)	9	7
**3h**	21	17	20	19	28 (4.68)	26 (18.75)
**3i**	9	7	10	7	5	8
**3j**	24 (9.37)	23 (4.68)	21 (9.37)	20 (18.75)	21 (18.75)	19 (37.5)
**3k**	7	9	12	10	13 (18.75)	14 (37.5)
**1**	6	5	8	6	-	-
**2**	7	8	9	7	-	-
Penicillin	33 (2.34)	35 (1.17)	29 (9.37)	28 (9.37)	-	-
Itrazole	-	-	-	-	31 (1.17)	28 (9.37)

*^a^* Standard solutions of 150 μg/mL were used to find the Zone of Inhibition; *^b^* for the selected compounds Minimum Inhibitory Concentration (MIC) values are given in brackets.

**Table 3:** 2-Hydroxy-3-chrysino dithiocarbamate derivatives (**3a**–**k**) demonstrating highest affinity (Moldock score) against *E. coli* FabH (pdb id: **1HNJ**) as predicted by molecular docking.

S. No	Ligand	Moldock Score [Grid](kcal/mol)	Moldock Score	Rerank Score	RMSD
1	Penicillin	−140.42	−141.09	−97.99	51.88
2	Chrysin	−95.05	−97.72	−24.61	44.38
3	**2**	−100.46	−97.65	−76.56	53.37
4	**3a**	−137.01	−140.03	−125.10	53.53
5	**3b**	−139.01	−139.60	−100.94	55.87
6	**3c**	−135.46	−136.85	−118.25	53.98
7	**3d**	−156.02	−158.49	−100.42	49.82
8	**3e**	−146.08	−146.70	−130.27	53.84
9	**3f**	−152.07	−153.97	−130.76	59.85
10	**3g**	−151.49	−155.46	−132.91	51.90
11	**3h**	−148.68	−146.88	−110.41	50.82
12	**3i**	−128.11	−133.98	−47.94	50.36
13	**3j**	−154.52	−157.49	−102.96	43.40
14	**3k**	−137.98	−139.31	−108.02	53.31

**Table 4:** 2-Hydroxy-3-chrysino dithiocarbamate derivatives (**3a**–**k**) demonstrating highest affinity (Moldock score) against *S. cerevisiae* (pdb id: **5EQB**) as predicted by molecular docking.

S. No	Ligand	Moldock Score [Grid](kcal/mol)	Moldock Score	Rerank Score	RMSD
1	Itrazole	−213.68	−218.38	−171.75	38.25
2	Chrysin	−96.56	−94.56	−81.26	38.67
3	**2**	−116.34	−115.26	−97.75	32.62
4	**3a**	−157.20	−154.09	−127.09	35.42
5	**3b**	−169.85	−170.41	−140.99	35.93
6	**3c**	−161.94	−164.89	−134.37	29.90
7	**3d**	−184.72	−186.61	−154.64	29.64
8	**3e**	−167.83	−170.79	−143.70	30.06
9	**3f**	−186.76	−189.74	−155.25	36.50
10	**3g**	−176.23	−173.46	−128.58	38.73
11	**3h**	−190.54	−192.50	−157.04	29.60
12	**3i**	−160.31	−161.26	−116.69	36.54
13	**3j**	−187.92	−189.65	−141.46	28.25
14	**3k**	−165.14	−164.57	−140.99	36.04

**Table 5:** Pharmacokinetic properties of chrysin, its 2-hydroxy-3-chrysino dithiocarbamate derivatives (**3a**–**k**) and standard drug penicillin.

Comp	Gpcr Ligand	Ion Channel Modulator	Kinase Inhibitor	Nuclear Receptor Ligand	Protease Inhibitor	Enzyme Inhibitor	m_i_logP [a]	TPSA (A^2^) [b]	n Violation [c]	M.wt [d]	nON [e]	nOHNH [f]	%ABS	MV [g]
**Rule**							≤5	---	≤1	<500	<10	<5		
**Penicillin**	0.01	−0.42	−0.71	−0.37	0.86	0.30	1.82	86.71	0	334.40	6	2	79.08	287.55
**Chrysin**	−0.11	−0.08	0.15	0.30	−0.30	0.26	2.94	70.67	0	254.24	4	2	84.61	216.03
**2**	−0.03	−0.35	0.26	0.21	0.03	0.21	3.31	72.20	0	310.31	5	1	84.09	265.57
**3a**	−0.12	−0.54	−0.31	−0.21	−0.28	0.07	4.70	83.14	0	471.60	6	2	80.31	407.39
**3b**	−0.12	−0.54	−0.29	−0.19	−0.26	0.01	4.20	83.14	0	457.57	6	2	80.31	390.59
**3c**	−0.18	−0.61	−0.29	−0.24	−0.31	0.04	3.64	92.37	0	473.57	7	2	77.13	399.57
**3d**	−0.11	−0.62	−0.27	−0.25	−0.26	0.01	5.08	86.38	2	562.71	7	2	79.19	491.58
**3e**	−0.14	−0.55	−0.29	−0.24	−0.28	0.08	4.18	83.14	0	489.64	6	2	80.31	408.71
**3f**	−0.10	−0.69	−0.28	−0.27	−0.27	−0.04	5.25	86.38	2	580.70	7	2	79.18	496.51
**3g**	−0.04	−0.51	−0.13	−0.27	−0.26	0.09	4.48	99.27	1	549.67	8	2	74.75	470.62
**3h**	−0.14	−0.71	−0.30	−0.30	−0.32	−0.09	5.44	95.61	2	578.71	8	2	76.01	500.32
**3i**	−0.09	−0.48	−0.23	−0.13	−0.20	0.02	4.30	92.37	1	501.63	7	2	77.13	432.75
**3j**	−0.03	−0.50	−0.27	−0.12	−0.06	0.09	4.72	112.68	1	572.71	9	2	70.12	497.52
**3k**	−0.17	−0.60	−0.34	−0.24	−0.35	0.04	4.55	83.14	0	459.59	6	2	80.31	400.95

[a] Calculated lipophilicity. [b] Total polar surface area. [c] No. of violations from Lipinski’s rule of five. [d] Molecular weight. [e] No. of hydrogen bond acceptors. [f] No. of hydrogen bond donors. [g] Molar volume.

## Supplementary Materials

Supporting information available online, includes ^1^H NMR, ^13^C NMR, and HRMS-ESI spectra of the synthesized compounds.
