# Supplementary material for: Molecular Design, Synthesis, and Biological Evaluation of 2-Hydroxy-3-Chrysino Dithiocarbamate Derivatives
Source: Molecules. 2019 Aug 21;24(17):3038. doi: 10.3390/molecules24173038 (PMC6749404; doi:10.3390/molecules24173038)
Supplement: Supplementary file 1 [file molecules-24-03038-s001.pdf]

Molecular design, synthesis and biological evaluation of 2-hydroxy-3-chrysino dithiocarbamate derivatives

Pulabala Ramesh [1,2], Vankadari Srinivasa Rao [2], Yi-An Hong[3], P. Muralidhar Reddy [2*] and Anren Hu[3*]

[1] Department of Chemistry, SR&BGNR. Government College (A), Khammam 507 002, India; rameshkuchem@gmail.com (PR)

[2]Department of Chemistry, Osmania University, Hyderabad 500 007, India; pmdreddy@gmail.com (PMR); chem.srinu44@gmail.com (VSR)

[3]Department of Laboratory Medicine and Biotechnology, College of Medicine, Tzu-Chi University, Hualien, Taiwan; amyhung840809@gmail.com (YH); anren@gms.tcu.edu.tw (AH);

***Corresponding author(s):** pmdreddy@gmail.com (PMR); Tel: +91-9848792423, anren@gms.tcu.edu.tw (AH) Tel.: +886-3-8565301 (ext. 2334 or 2335); Fax: +886-3-8571917 (AH.).

Supporting information

Synthesis of 5-hydroxy-7-(oxiran-2-ylmethoxy)-2-phenyl-4*H*-chromen-4-one (2): To a solution of chrysin **1** (4 mmol) in DMF (12 ml), was added K_2CO_3 (20 mmol) and the mixture was stirred for 20 min at room temperature. Epichlorohydrin (20 mmol) was then added drop wise to above mixture. The reaction mixture was heated at 50 °C for 6 h. After completion of reaction, the mixture was poured into the ice water. The precipitate and the extractions were combined and subjected to column chromatography (silica gel; eluent: PE:EA = 10:1) to afford **2** as pale yellow solid (780 mg, 64%).

m.p: 165-167 °C; ^{1}H NMR (500MHz, $CDCl_3$) δ 12.73 (s, 1H), 7.89-7.86 (m, 2H), 7.53 (dd, J = 17.2, 6.6 Hz, 3H), 6.67 (s, 1H), 6.53 (d, J = 2.2 Hz, 1H), 6.38 (d, J = 2.2 Hz, 1H0, 4.34 (dd, J = 11.0, 2.9 Hz, 1H), 4.01 (dd, J = 11.0, 5.9 Hz, 1H), 3.39 (dt, J = 6.8, 2.8 Hz, 1H), 2.98-2.93 (m, 1H), 2.79 (dd, J = 4.8, 2.6 Hz, 1H0; ^{13}C NMR (125 MHz, $CDCl_3$) δ 182.4, 164.3, 164.07, 162.2, 157.72, 131.8, 131.2, 129.8, 126.3, 106.0, 105.9, 98.6, 93.3, 69.2, 49.2, 44.5; HRMS (ESI): *m/z* calcd. for $C_{18}H_{14}O_5$ $[M+H]^+$ 311.0919, found 311.0920.

Typical Procedure for the synthesis of 3: To a solution of secondary amine (1.5 equiv.) in ACN, CS_2 (3 equiv.) was added drop wise. The reactions mixture was then stirred at room temperature for 30 min. To this reaction mixture, **2** (1 equiv.) and LiBr (0.04 eqiv.) were added and then stirred at 60 °C for appropriate time as monitored by TLC. After completion, the reaction mixture was diluted with ice cold water and extracted with EtOAc. Then, evaporation of EtOAc gave a crude residue which was further purified by column chromatography (silica gel, ethyl acetate/hexane as eluent).

2-Hydroxy-3-((5-hydroxy-4-oxo-2-phenyl-4*H*-chromen-7-yl)oxy) propyl piperidine-1-carbodithioate (3a): m.p: 134-136 °C; ^{1}H NMR (500 MHz, $CDCl_3$) δ 12.71 (s, 1H), 7.89 (dd, J = 8.1, 1.5 Hz, 2H), 7.59-7.49 (m, 3H), 6.67 (s, 1H), 6.56 (d, J = 2.2 Hz, 1H), 6.41 (d, J = 2.2 Hz, 1H), 4.40-4.35 (m, 1H), 4.31 (s, 2H), 4.15 (ddd, J = 15.3, 9.5, 5.4 Hz, 1H), 3.95 (s, 2H), 3.85 (dd, J = 14.6, 4.3 Hz, 2H), 3.68 (dd, J = 14.6, 6.8 Hz, 2H), 3.28 (d, J = 4.4 Hz, 1H), 1.73 (br.s, 6H); ^{13}C NMR (125 MHz, $CDCl_3$) δ 195.5, 182.4, 164.4, 164.1, 162.2, 157.7, 131.8, 131.3, 129.1,

126.3, 106, 105.9, 98.8, 93.2, 71, 69.5, 39.5, 24.2; HRMS (ESI): *m/z* calcd. for $C_{24}H_{25}NO_5S_2$ $[M+H]^+$ 472.1252, found 472.1258.

2-Hydroxy-3-((5-hydroxy-4-oxo-2-phenyl-4*H*-chromen-7-yl)oxy)propyl pyrrolidine-1-carbodithioate (3b):
m.p: 144-146 ºC; ^{1}H NMR (500 MHz, $CDCl_3$) δ 12.71 (s,1H), 7.89 (dd, J = 8.1, 1.5 Hz, 2H), 7.59-7.49 (m, 3H), 6.67 (s, 1H), 6.56 (d, J = 2.2 Hz, 1H), 6.41 (d, J = 2.2 Hz, 1H), 4.40-4.35 (m, 1H), 4.31 (s, 2H), 4.15 (ddd, J = 15.3, 9.5, 5.4 Hz, 1H), 3.95 (s, 2H), 3.85 (dd, J = 14.6, 4.3 Hz, 2H), 3.68 (dd, J = 14.6, 6.8 Hz, 2H), 3.28 (d, J = 4.4 Hz, 1H), 1.73 (br.s, 6H); ^{13}C NMR (125 MHz, $CDCl_3$) δ 195.5, 182.4, 164.4, 164.1, 162.2, 157.7, 131.8, 131.3, 129.1, 126.3, 106, 105.9, 98.8, 93.2, 71, 69.5, 39.5, 24.2; HRMS (ESI): *m/z* calcd. for $C_{23}H_{23}NO_5S_2$ $[M+H]^+$ 458.1096, found 458.1106.

2-Hydroxy-3-((5-hydroxy-4-oxo-2-phenyl-4*H*-chromen-7-yl)oxy)propyl morpholine-4-carbodithioate (3c):
m.p: 146-150 ºC; ^{1}H NMR (500 MHz, $CDCl_3$) δ 12.71 (s, 1H), 7.89 (dd, J = 8.1, 1.5 Hz, 2H), 7.57-7.50 (m, 3H), 6.68 (s, 1H), 6.55 (d, J = 2.2 Hz, 1H), 6.40 (d, J = 2.2 Hz, 1H), 4.42-4.35 (m, 2H), 4.15 (ddd, J = 15.4, 9.6, 5.3 Hz, 1H), 4.02 (br.s., 2H), 3.85 (dd, J = 14.5, 4.3 Hz, 1H), 3.79 (s, 4H), 3.69 (dd, J = 14.5, 7.0 Hz, 1H); ^{13}C NMR (125 MHz, $CDCl_3$) δ 197.5, 182.5, 164.3, 164.1, 162.2, 157.7, 131.9, 131.3, 129.1, 126.3, 106.0, 105.9, 98.7, 93.2, 71.0, 69.3, 39.4. HRMS (ESI): *m/z* calcd. for $C_{23}H_{23}NO_6S_2$ $[M+H]^+$ 474.1045, found 474.1043.

2-Hydroxy-3-((5-hydroxy-4-oxo-2-phenyl-4*H*-chromen-7-yl)oxy)propyl 4-benzylpiperazine-1-carbodithiote (3d):
m.p: 154-156 ºC; ^{1}H NMR (500 MHz, $CDCl_3$) δ 12.71 (s, 1H), 7.89 (dd, J = 8.1, 1.5 Hz, 2H), 7.59-7.49 (m, 3H), 7.37-7.30 (m, 5H), 6.68 (s, 1H), 6.55 (d, J = 2.2 Hz, 1H), 6.40 (d, J = 2.2 Hz, 1H), 4.37 (s, 3H), 4.15 (ddd, J = 15.4, 9.5, 5.3 Hz, 1H), 3.99 (s, 2H), 3.84 (dd, J = 14.6, 4.3 Hz, 1H), 3.67 (dd, J = 14.6, 6.9 Hz, 1H), 3.55 (s, 2H), 3.18 (s, 1H), 2.56 (br.s., 4H); ^{13}C NMR (125 MHz, $CDCl_3$) δ 196.6, 182.4, 164.4, 164.1, 162.2, 157.7, 137.2, 131.8, 131.3, 129.1, 128.4, 127.4, 126.3, 106, 105.9, 98.8, 93.2, 71, 69.4, 62.4, 52.3, 39.5; HRMS (ESI): *m/z* calcd. for $C_{30}H_{30}N_2O_5S_2$ $[M+H]^+$ 563.1674, found 563.1686.

2-Hydroxy-3-((5-hydroxy-4-oxo-2-phenyl-4*H*-chromen-7-yl)oxy)propyl thiomorpholine-4-carbodithioate (3e):
m.p: 168-170 ºC; ^{1}H NMR (500 MHz, $CDCl_3$) δ 12.72 (s, 1H), 7.89 (dd, J = 8.1, 1.5 Hz, 2H), 7.58-7.50 (m, 3H), 6.68 (s, 1H), 6.56 (d, J = 2.2 Hz, 1H), 6.40 (d, J = 2.2 Hz, 1H), 4.64 (br.s, 2H), 4.41-4.34 (m, 2H), 4.32 (br.s, 1H), 4.15 (ddd, J = 15.4, 9.5, 5.3 Hz, 1H), 3.85 (dd, J = 14.5, 4.3 Hz, 1H), 3.68 (dd, J = 14.5, 6.9 Hz, 1H), 3.07 (d, J = 4.5 Hz, 1H), 2.78 (s, 4H); ^{13}C NMR (125 MHz, $CDCl_3$+DMSO-d_6) δ 196.7, 182.4, 164.5, 164.1, 162.1, 157.7, 131.8, 131.2, 129.1, 126.3, 105.9, 105.8, 98.8, 93.2, 71.2, 68.9, 29.6, 27.2; HRMS (ESI): *m/z* calcd. for $C_{23}H_{23}NO_5S_3$ $[M+H]^+$ 490.0817, found 490.0822.

2-Hydroxy-3-((5-hydroxy-4-oxo-2-phenyl-4H-chromen-7-yl)oxy)propyl 4-(4-fluorophenyl) piperazine-1-carbodithiate (3f):
m.p: 146-148 ºC; ^{1}H NMR (500 MHz, $CDCl_3$) δ 12.72 (s, 1H), 7.89 (d, J = 8.3 Hz, 2H), 7.54 (m, 3H), 7.03-6.95 (m, 2H), 6.88 (dd, J = 9.1, 4.5 Hz, 2H), 6.68 (s, 1H), 6.56 (d, J = 2.1 Hz, 1H), 6.41 (d, J = 2.2 Hz, 1H), 4.52 (br.s, 2H), 4.45-4.36 (m, 1H), 4.16 (ddd, J = 15.3, 9.5, 5.3 Hz, 1H), 3.86 (dd, J = 14.6, 4.3 Hz, 1H), 3.70 (dd, J = 14.6, 4.3 Hz, 1H), 3.26-3.18 (m, 4H), 3.13 (d, J = 4.5 Hz, 1H); ^{13}C NMR (125 MHz, $CDCl_3$) δ 197.2, 182.4, 164.3, 164.1, 162.2, 158.7, 157.7, 156.8, 146.88 (d, J = 2.4 Hz), 131.8, 131.3, 129.1, 126.3, 118.46 (d, J = 7.8 Hz), 115.9, 115.7, 106, 105.9, 98.7, 93.2, 71, 69.3, 49.9, 39.3; HRMS (ESI): *m/z* calcd. for $C_{29}H_{27}FN_2O_5S_2$ $[M+H]^+$ 567.1424, found 567.1439.

2-Hydroxy-3-((5-hydroxy-4-oxo-2-phenyl-4*H*-chromen-7-yl)oxy)propyl 4-(pyridine-2-yl) piperazine-1-carbodithioate (3g)

m.p: 152-154 ºC; ^{1}H NMR (500 MHz, $CDCl_3$) δ 12.71 (s, 1H), 8.20 (dd, J = 4.9, 1.2 Hz, 1H), 7.89 (dd, J = 8.0, 1.5 Hz, 2H), 7.59-7.48 (m, 4H), 6.71-6.69 (m, 1H), 6.68 (d, J = 2.5 Hz, 1H), 6.63 (d, J = 1H), 6.56 (d, J = 2.1 Hz, 1H), 6.41 (d, J = 2.2 Hz, 1H), 4.49 (s, 1H), 4.40 (s, 1H), 4.18 (td, J = 11.9, 6.0 Hz, 2H), 4.15 (ddd, J = 15.3, 9.5, 5.4 Hz, 1H), 3.86 (dt, J = 18.6, 9.3 Hz, 1H), 3.70 (m, 6H), 3.19 (s, 1H); ^{13}C NMR (125 MHz, $CDCl_3$ + DMSO-d_6) δ 196.6, 182.3, 164.9, 164, 161.9, 158.4, 157.7, 147.8, 137.7, 131.9, 131.1, 129.1, 126.3, 113.8, 107, 105.7, 105.6, 98.9, 93.3, 71.8, 68.1, 44.2; HRMS (ESI): *m/z* calcd. for $C_{28}H_{27}N_3O_5S_2$ $[M+H]^+$ 550.1470, found 550.1492.

2-Hydroxy-3-((5-hydroxy-4-oxo-2-phenyl-4*H*-chromen-7-yl)oxy)propyl 4-(4-methoxyphenyl) piperazine-1-carbodithioate (3h)

m.p: 151-153 ºC; ^{1}H NMR (500 MHz, $CDCl_3$) δ 12.71 (s, 1H), 7.88 (d, J = 8.3 Hz, 2H), 7.59-7.47 (m, 3H), 6.90 (d, J = 9.1 Hz, 2H), 6.85 (d, J = 9.1 Hz, 2H), 6.67 (s, 1H), 6.56 (d, J = 2.1 Hz, 1H), 6.41 (d, J = 2.2 Hz, 1H), 4.51 (br. s, 2H), 4.39 (dd, J = 10.2, 4.6 Hz, 1H), 4.16 (ddd, J = 15.3, 9.5, 5.3 Hz, 1H), 3.86 (dd, J = 14.6, 4.3 Hz, 1H), 3.77 (s, 3H), 3.70 (dd, J = 14.6, 6.9 Hz, 1H), 3.21-3.15 (m, 5H); ^{13}C NMR (125 MHz, $CDCl_3$) δ 197, 182.4, 164.4, 164.1, 162.2, 157.7, 154.6, 144.5, 131.8, 131.3, 129.1, 126.3, 118.9, 114.6, 106, 105.9, 98.8, 93.2, 71, 69.4, 55.6, 50.5, 39.6; HRMS (ESI): *m/z* calcd. for $C_{30}H_{30}N_2O_6S_2$ $[M+H]^+$ 579.1624, found 579.1627.

2-Hydroxy-3-((5-hydroxy-4-oxo-2-phenyl-4*H*-chromen-7yl)oxy)propyl cis-3,5-dimethylmorpholine-4-carbodithioate (3i)

m.p: 181-183 ºC; ^{1}H NMR (500 MHz, $CDCl_3$) δ 12.72 (s, 1H), 7.89 (dd, J = 8.1, 1.5 Hz, 2H), 7.63-7.43 (m, 3H), 6.68 (s, 1H), 6.55 (d, J = 2.2 Hz, 1H), 6.40 (d, J = 2.2 Hz, 1H), 5.45 (s, 1H), 4.51 (s, 1H), 4.44-4.33 (m, 1H), 4.15 (ddd, J = 15.4, 9.5, 5.3 Hz, 1H), 3.85 (dd, J = 14.5, 4.3 Hz, 1H), 3.68 (dd, J = 14.5, 6.9 Hz, 3H), 3.13 (d, J = 4.4 Hz, 1H), 2.95 (s, 1H), 2.79 (s, 1H), 1.26 (s, 3H), 1.25 (s, 3H); ^{13}C NMR (125 MHz, $CDCl_3$) δ 196.9, 182.4, 164.3, 164.1, 162.3, 157.7, 131.8, 131.3, 129.1, 126.3, 106, 105.9, 98.7, 93.2, 71, 69.3, 39.3, 18.5; HRMS (ESI): *m/z* calcd. for $C_{25}H_{27}NO_6S_2$ $[M+H]^+$ 502.1358, found 502.1366.

tert-Butyl 4-(((2-hydroxy-3-((5-hydroxy-4-oxo-2-phenyl-4*H*-chromen-7-yl)oxy)propyl)thio) carbonothioyl)piperazine-1-carboxylate (3j)

m.p: 140-142 ºC; ^{1}H NMR (500 MHz, $CDCl_3$) δ 12.72 (s, 1H), 7.89 (d, J = 6.6 Hz, 2H), 7.61-7.48 (m, 3H), 6.68 (s, 1H), 6.56 (d, J = 2.2 Hz, 1H), 6.40 (d, J = 2.2 Hz, 1H), 4.38 (dd, J = 10.5, 5.7 Hz, 2H), 4.15 (ddd, J = 15.4, 9.5, 5.3 Hz, 1H), 4.00 (br.s, 2H), 3.85 (dd, J = 14.6, 4.3 Hz, 1H), 3.68 (dd, J = 14.6, 7.0 Hz, 1H), 3.60-3.54 (m, 4H), 3.11 (d, J = 4.4 Hz, 1H), 1.48 (s, 9H); ^{13}C NMR (125 MHz, $CDCl_3$) δ 197.5, 182.4, 164.3, 164.1, 162.2, 157.7, 154.4, 131.6, 131.3, 129.1, 126.3, 106, 105.9, 98.7, 93.2, 80.7, 71, 69.3, 39.6, 28.3; HRMS (ESI): *m/z* calcd. for $C_{28}H_{32}N_2O_7S_2$ $[M+H]^+$ 573.1729, found 573.1731.

2-Hydroxy-3-((5-hydroxy-4-oxo-2-phenyl-4*H*-chromen-7-yl)oxy)propyl diethyl carbamodithioate (3k)

m.p: 158-160 ºC; ^{1}H NMR (500 MHz, $CDCl_3$) δ 12.71 (s, 1H), 7.89 (d, J = 6.7 Hz, 2H), 7.60-7.47 (m, 3H), 6.68 (s, 1H), 6.56 (d, J = 2.2 Hz, 1H), 6.40 (d, J = 2.2 Hz, 1H), 4.38 (dd, J = 11.2, 5.2 Hz, 1H), 4.15 (ddd, J = 15.3, 9.5, 5.4 Hz, 1H), 4.05 (dt, J = 10.8, 6.7 Hz, 2H), 3.82 (m, 3H), 3.67 (dd, J = 14.7, 6.8 Hz, 1H), 3.35 (d, J = 4.4 Hz, 1H), 1.33 (t, J = 7.1 Hz, 3H), 1.29 (t, J = 7.1 Hz, 3H); ^{13}C NMR (125 MHz, $CDCl_3$) δ 195.7, 182.4, 164.4, 164, 162.2, 157.7, 131.8, 131.3, 129, 126.3, 106, 105.9, 98.8, 93.2, 71, 69.5, 50.2, 47.1, 39.5, 12.5, 11.5; HRMS (ESI): *m/z* calcd. for $C_{23}H_{25}NO_5S_2$ $[M+H]^+$ 460.1252, found 460.1256.

Copies of Spectra

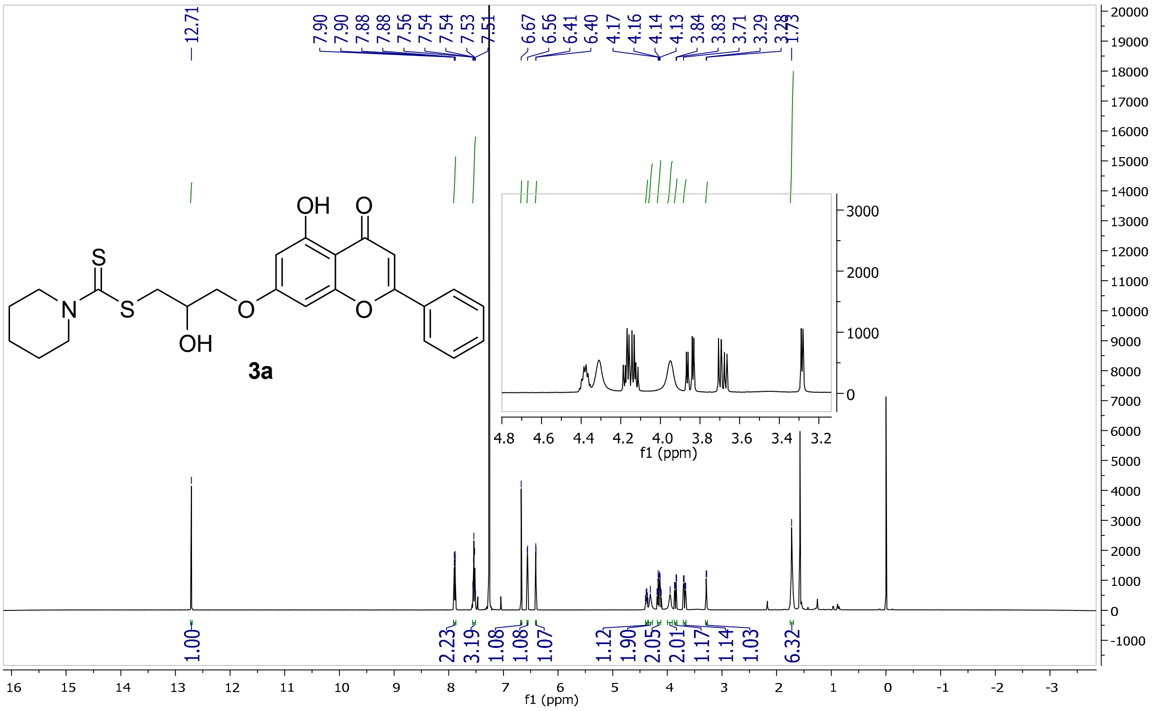

^{1}H NMR (500 MHz, $CDCl_3$) spectrum of compound **3a**

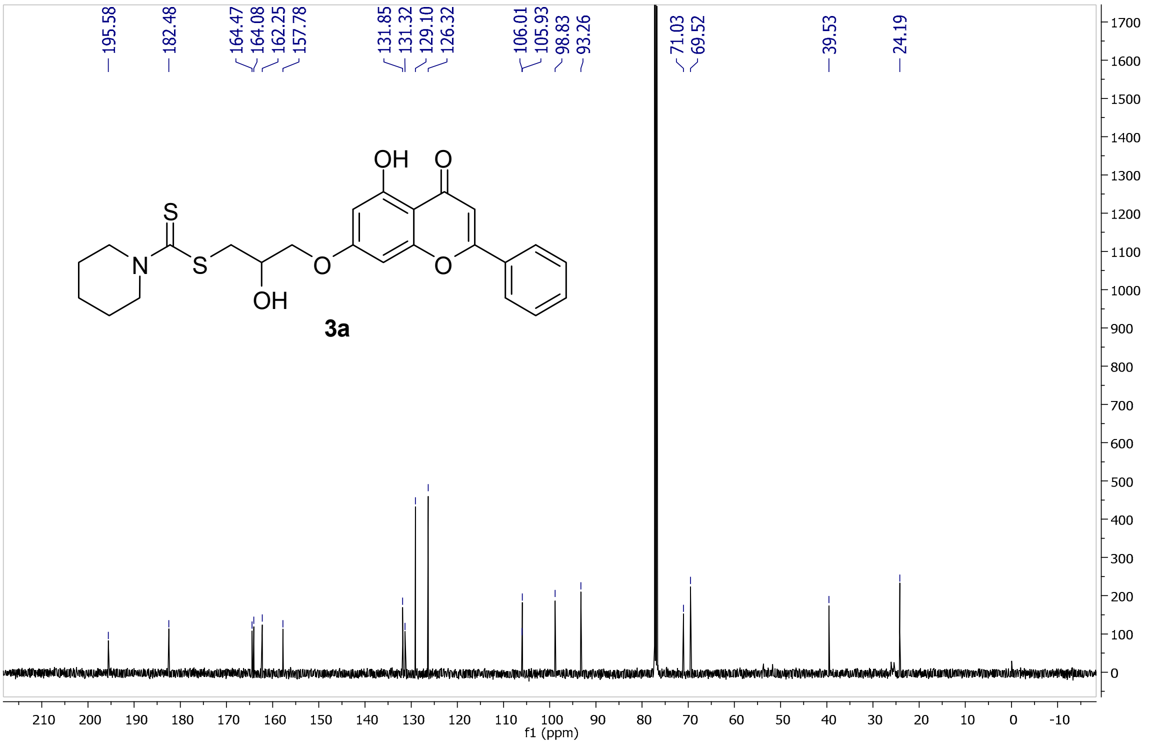

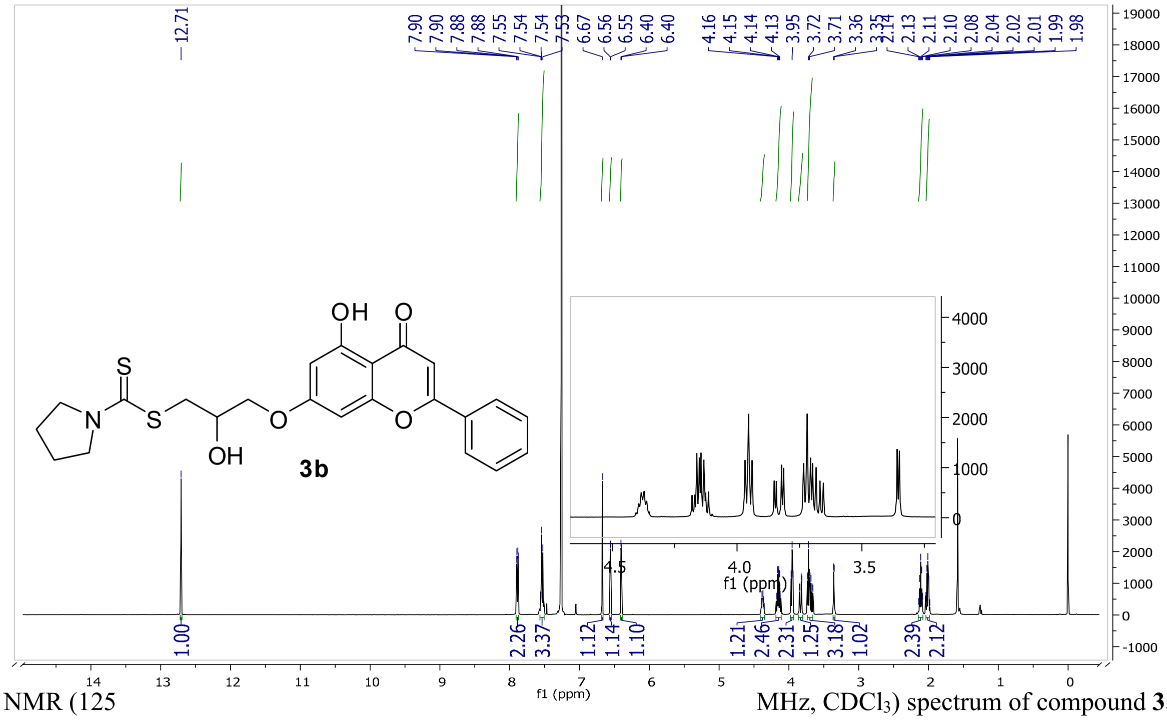

^{13}C NMR (125 MHz, $CDCl_3$) spectrum of compound **3a**

^{1}H NMR (500 MHz, $CDCl_3$) spectrum of compound **3b**

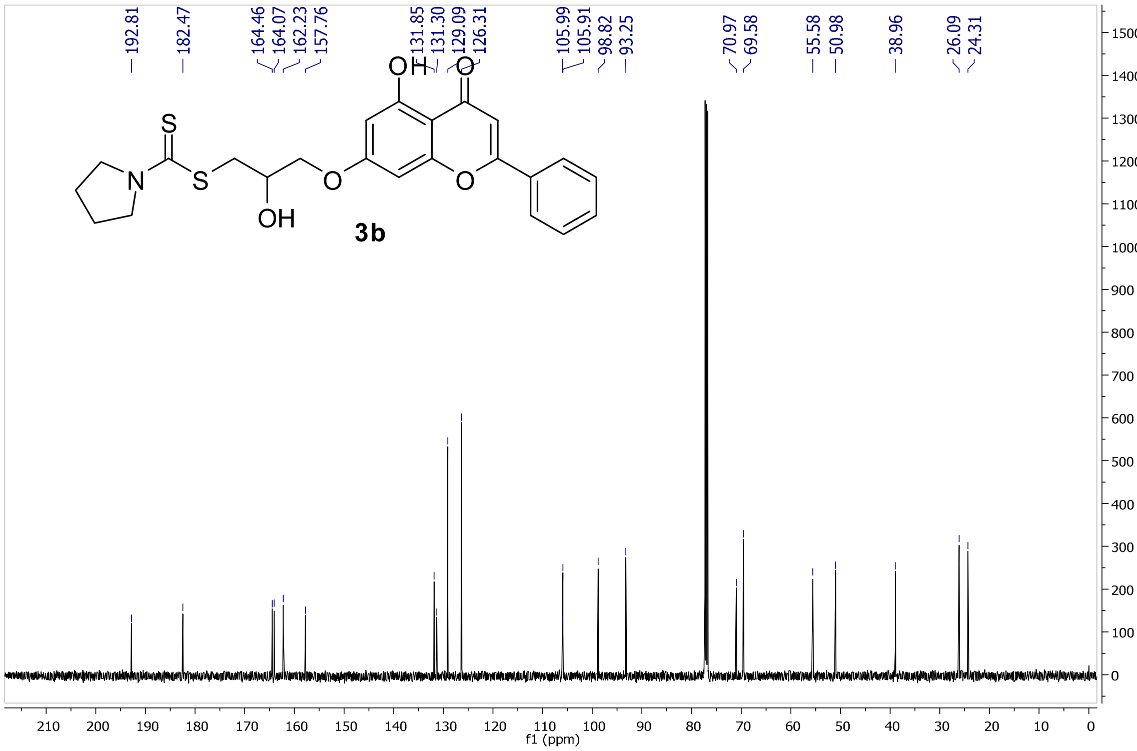

^{13}C NMR (125 MHz, $CDCl_3$) spectrum of compound **3b**

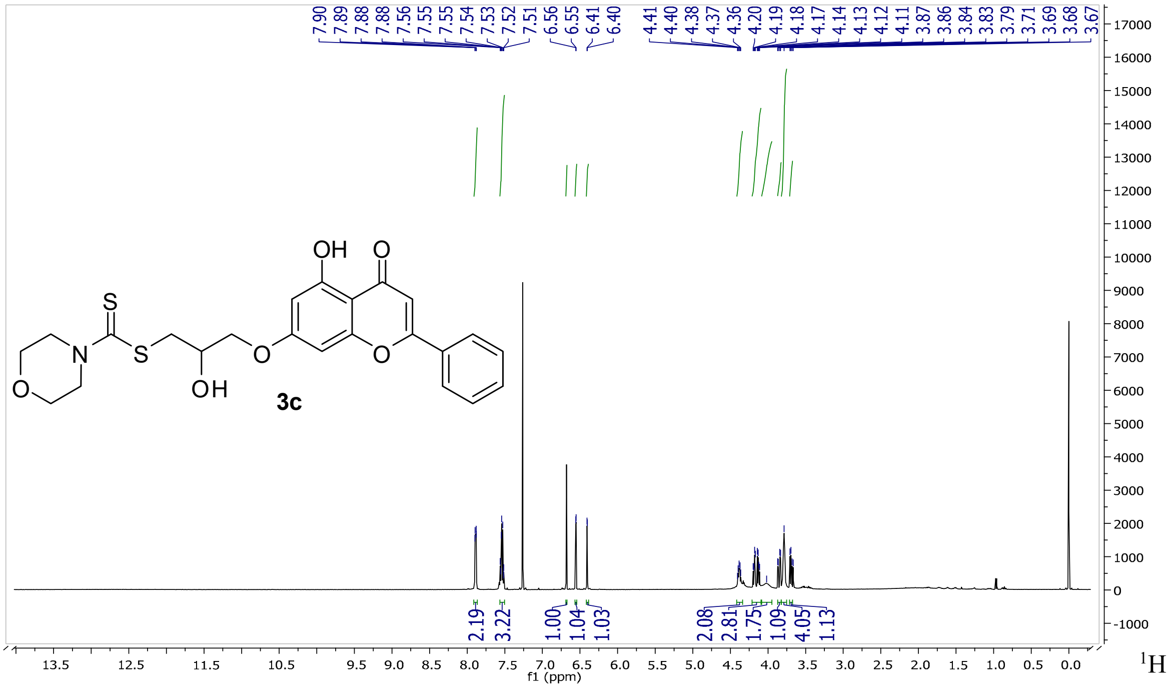

^1H NMR (500 MHz, $CDCl_3$) spectrum of compound **3c**

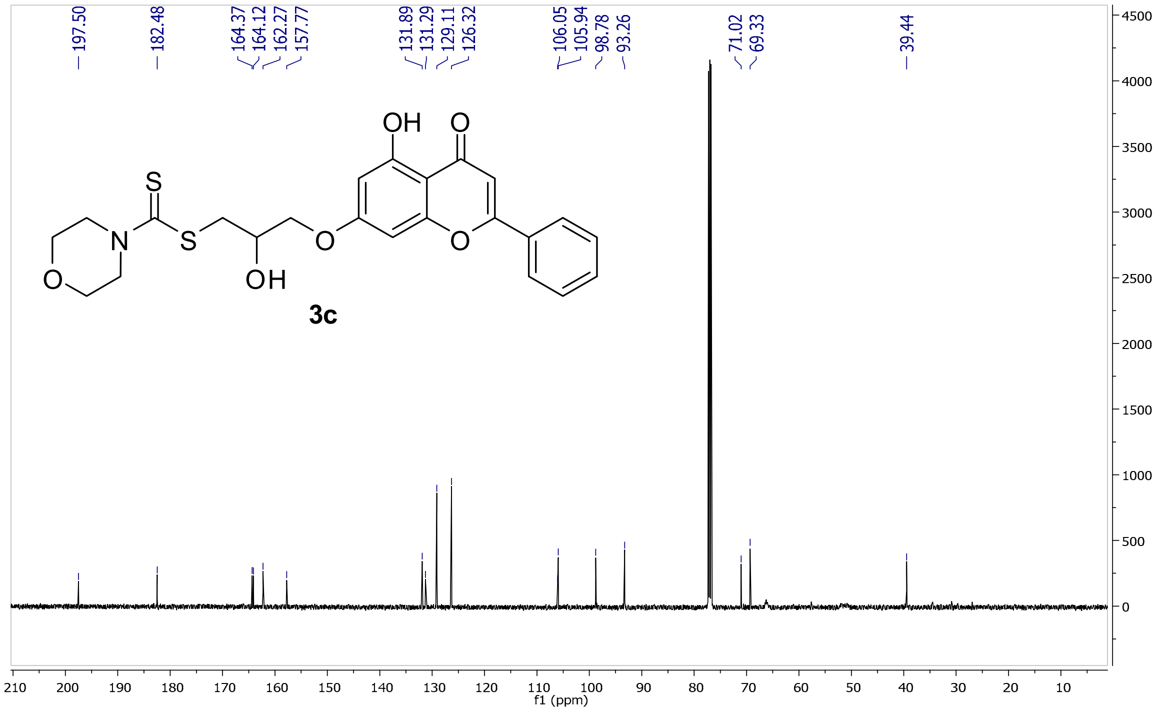

^{13}C NMR (125 MHz, $CDCl_3$) spectrum of compound **3c**

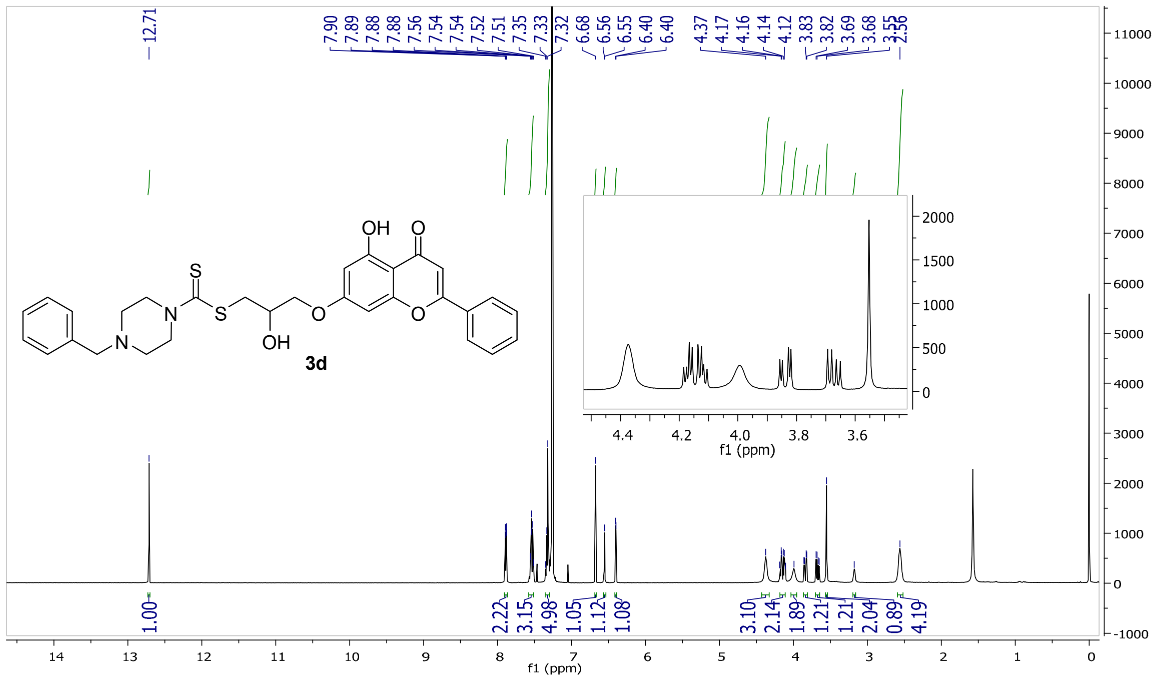

^{1}H NMR (500 MHz, $CDCl_3$) spectrum of compound **3d**

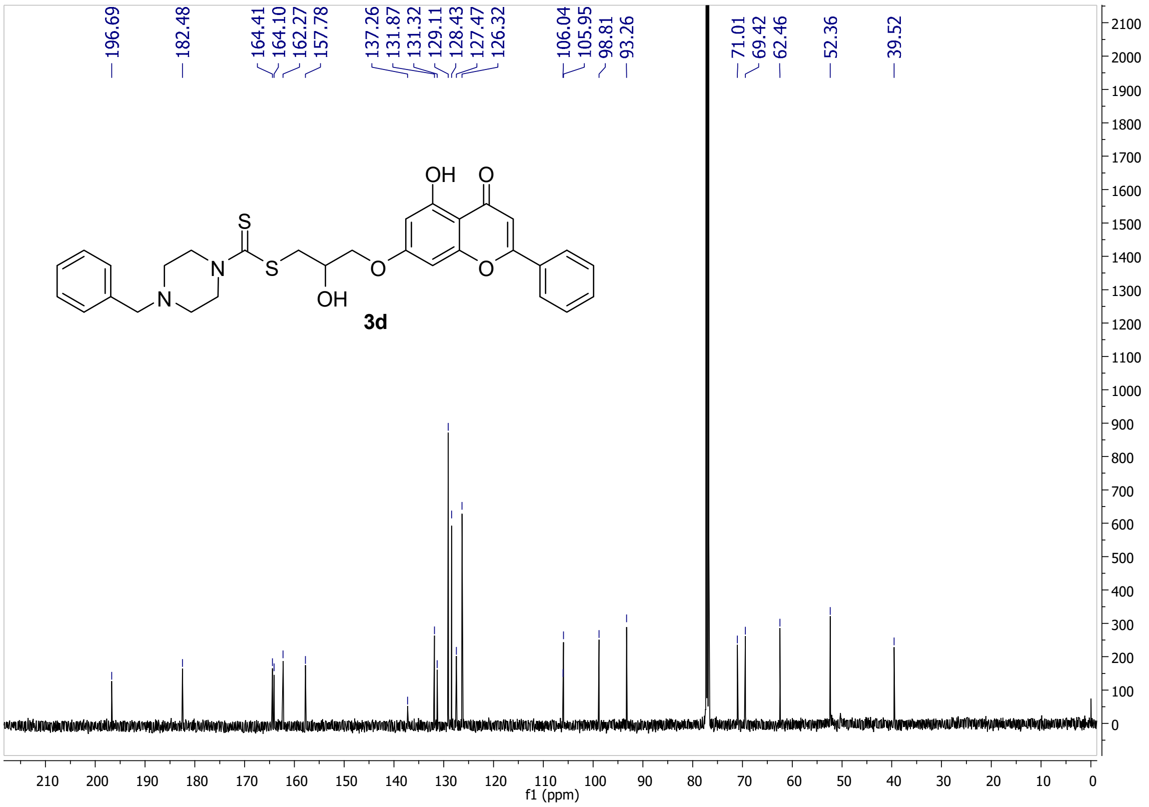

^{13}C NMR (125 MHz, $CDCl_3$) spectrum of compound **3d**

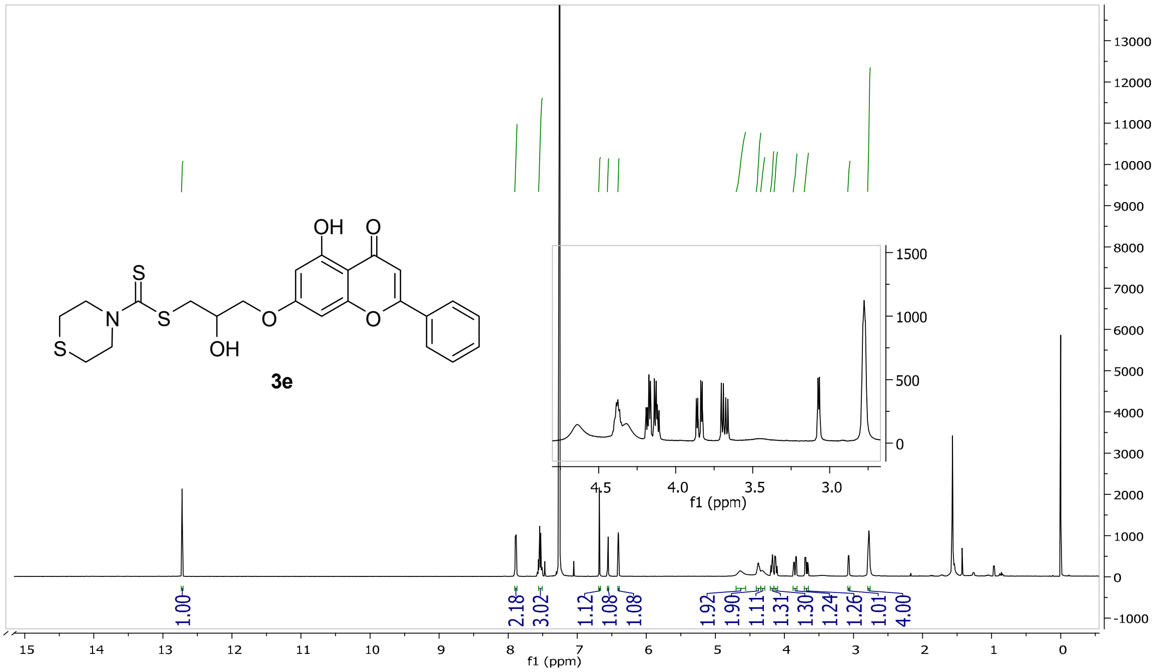

^{1}H NMR (500 MHz, $CDCl_3$) spectrum of compound **3e**

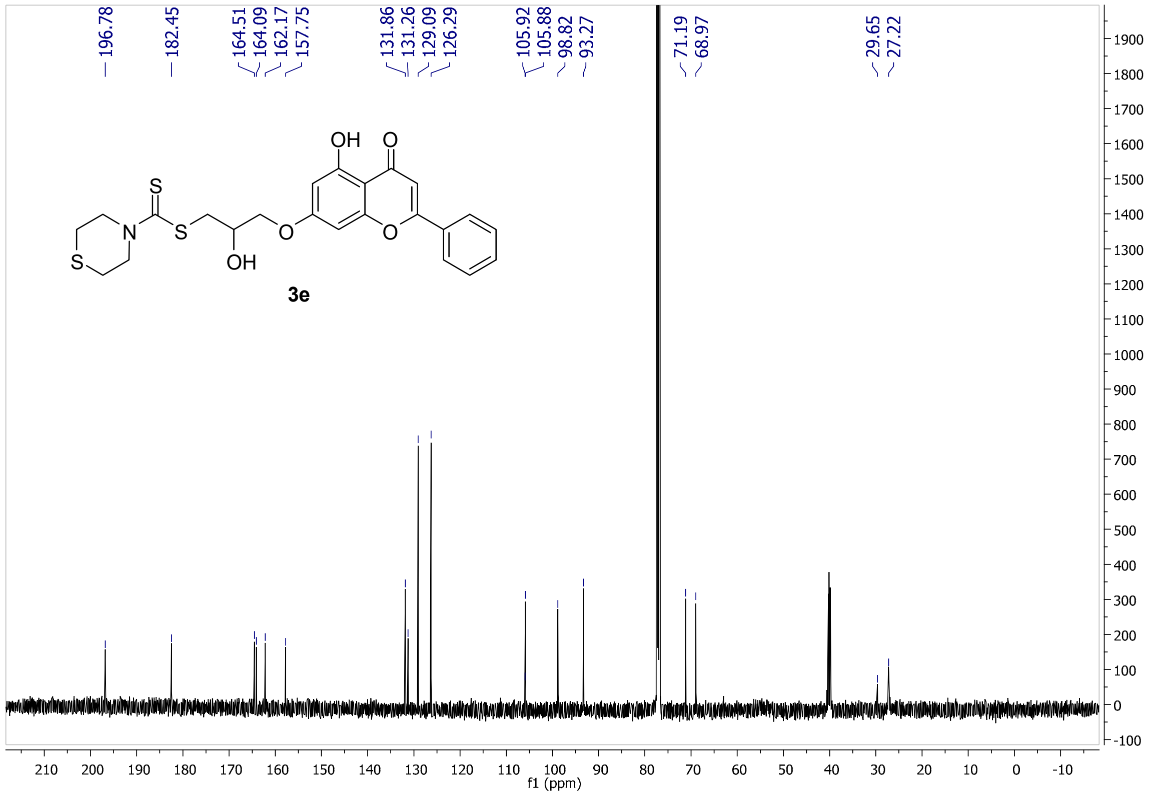

^{13}C NMR (125 MHz, $CDCl_3$+ DMSO-d_6) spectrum of compound **3e**

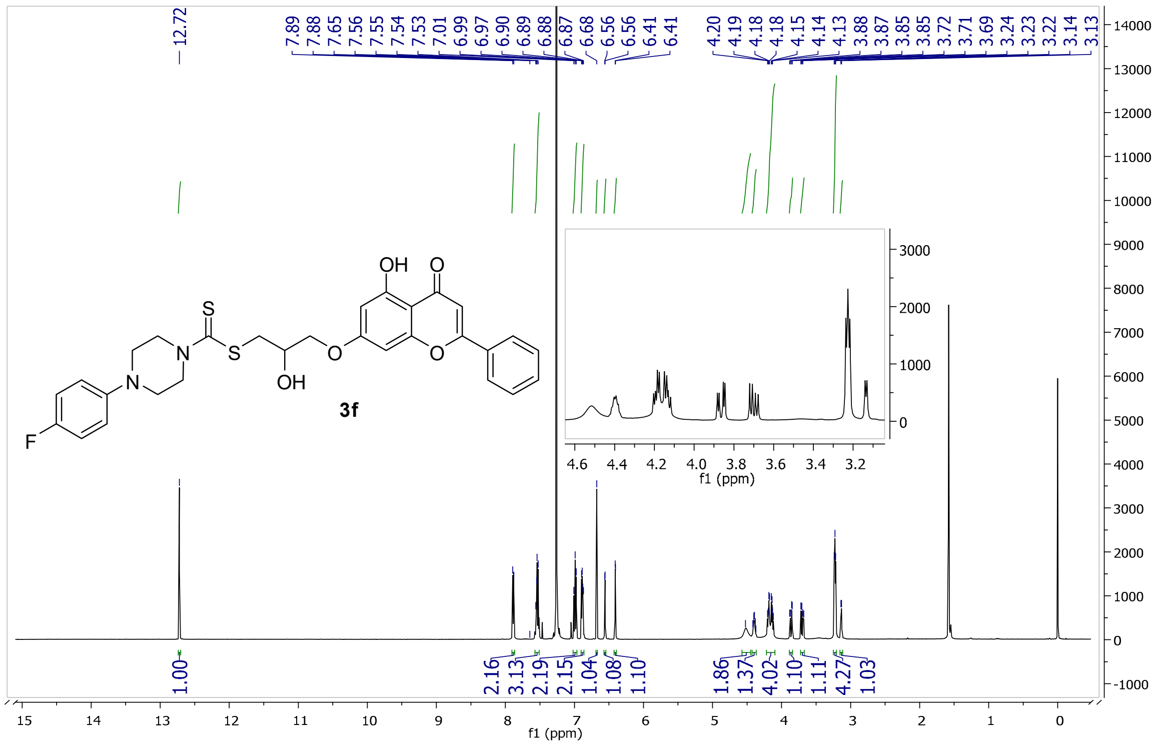

^{1}H NMR (500 MHz, $CDCl_3$) spectrum of compound **3f**

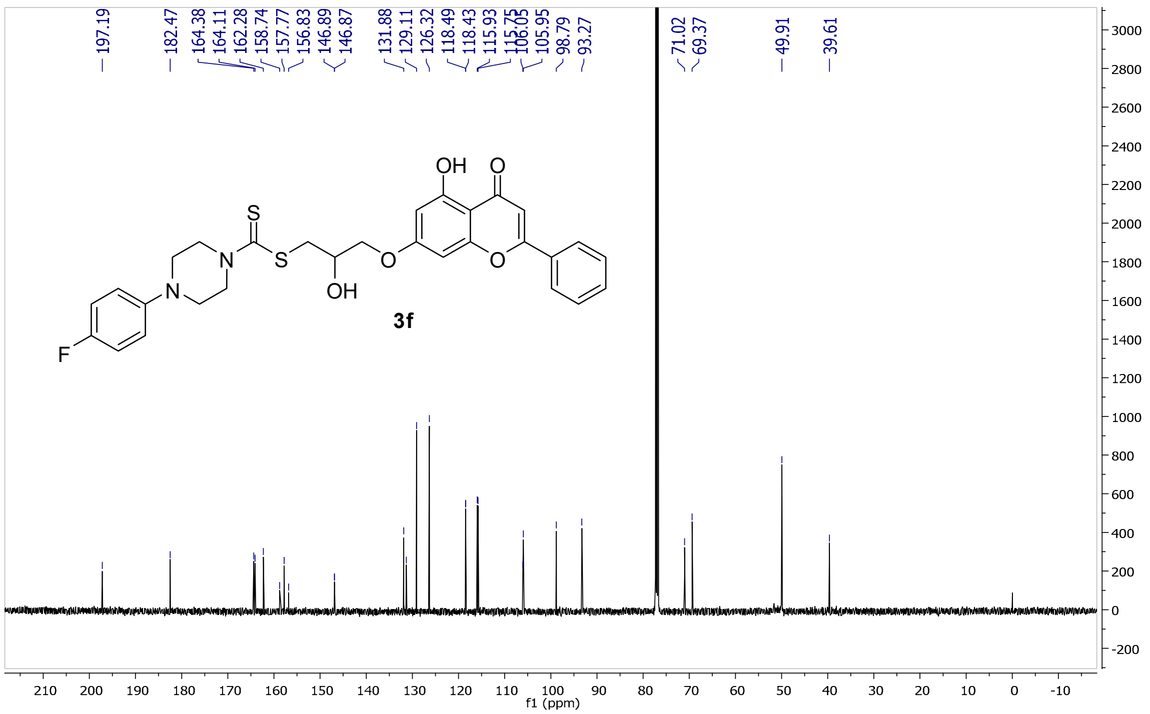

^{13}C NMR (125 MHz, $CDCl_3$) spectrum of compound **3f**

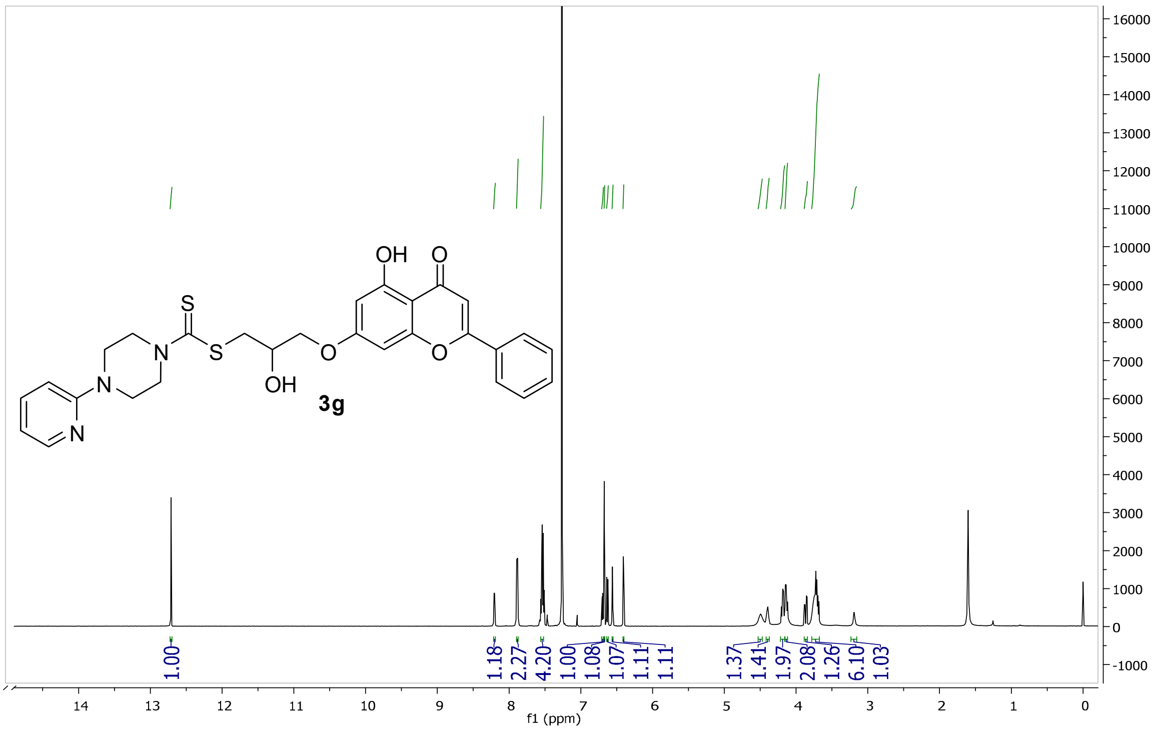

^{1}H NMR (500 MHz, $CDCl_3$) spectrum of compound **3g**

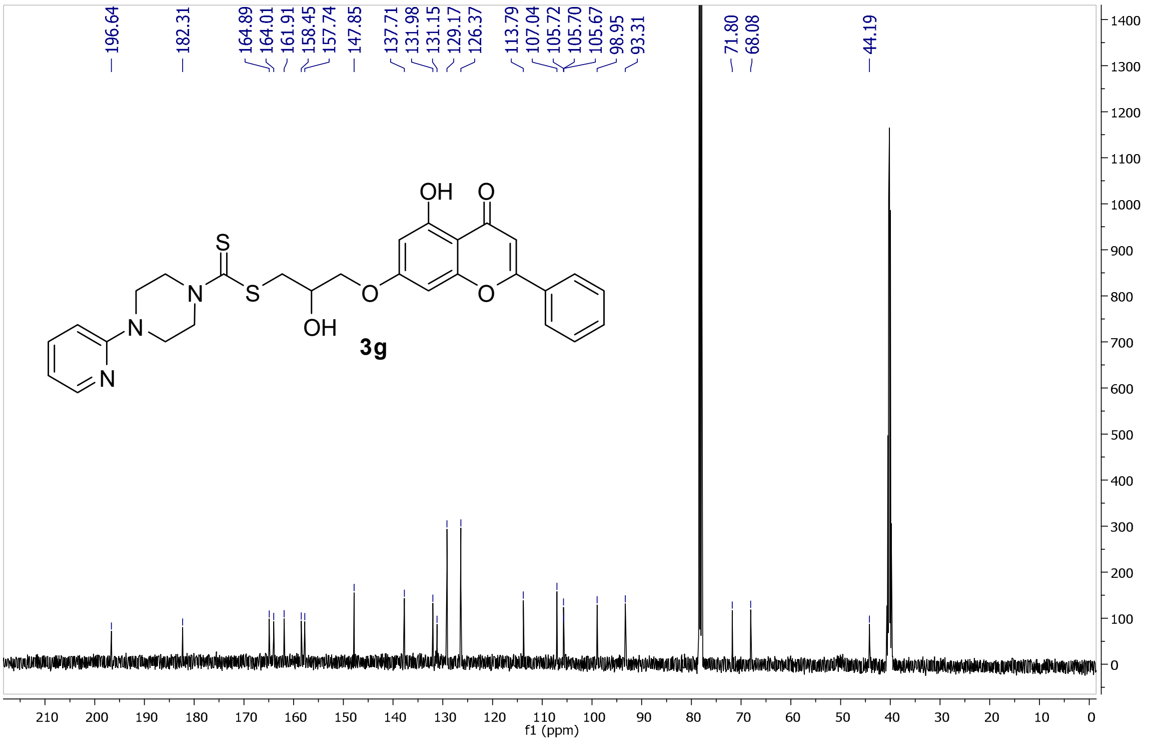

^{13}C NMR (125 MHz, $CDCl_3$+ DMSO-d_6) spectrum of compound **3g**

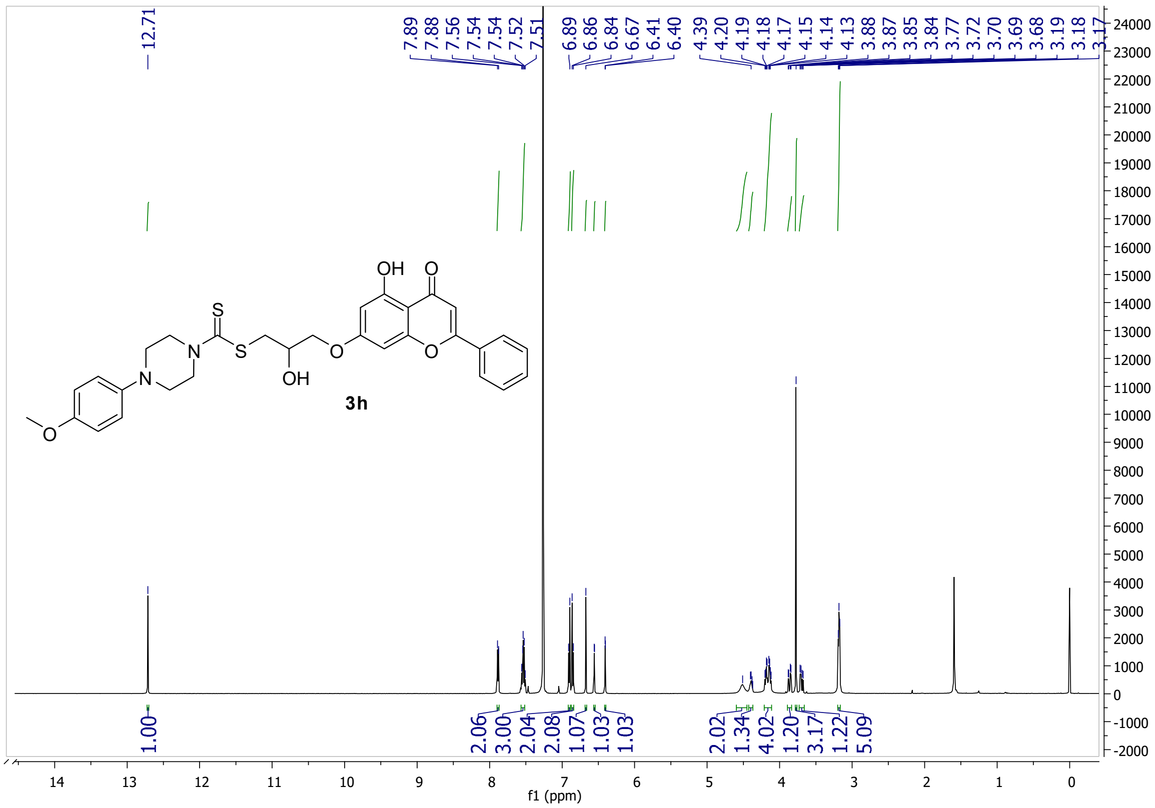

^{1}H NMR (500 MHz, $CDCl_3$) spectrum of compound **3h**

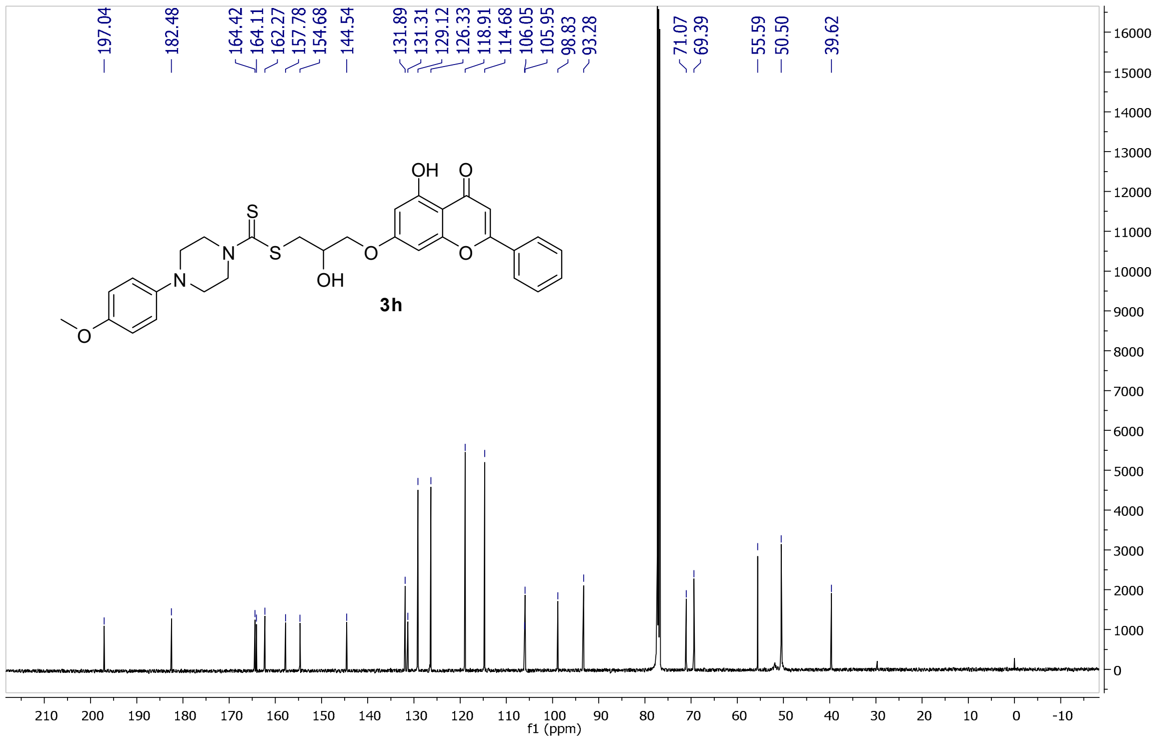

^{13}C NMR (125 MHz, $CDCl_3$) spectrum of compound **3h**

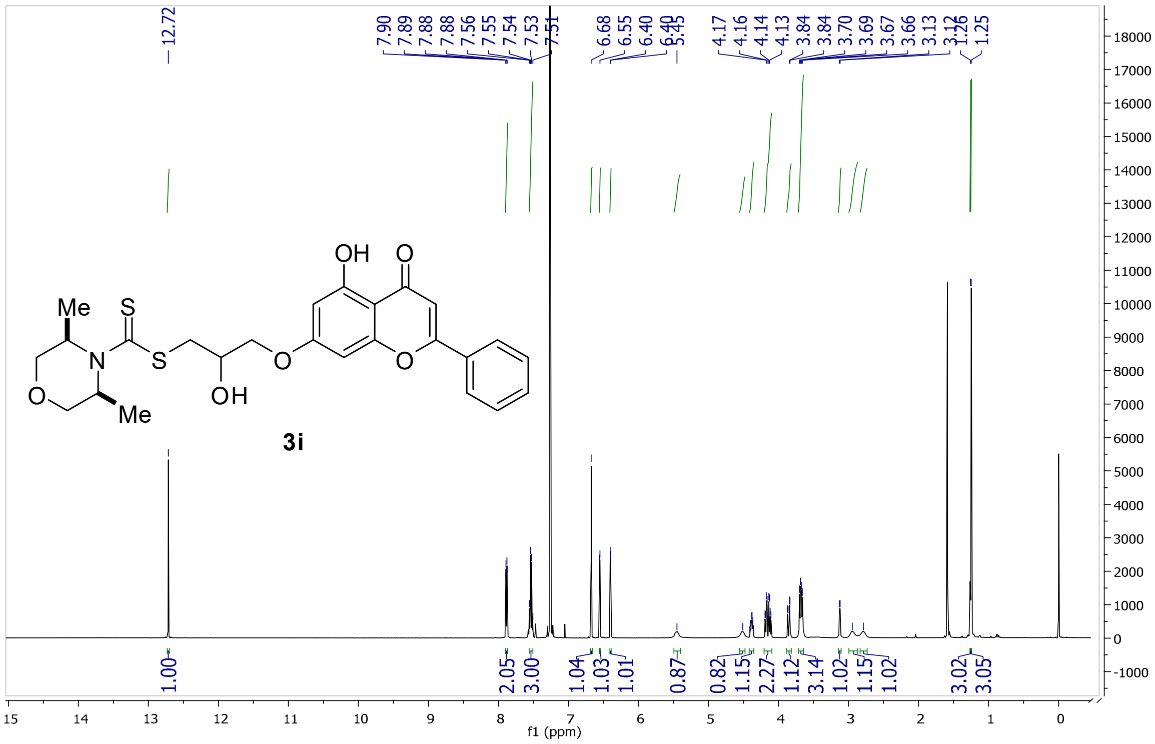

^{1}H NMR (500 MHz, $CDCl_3$) spectrum of compound **3i**

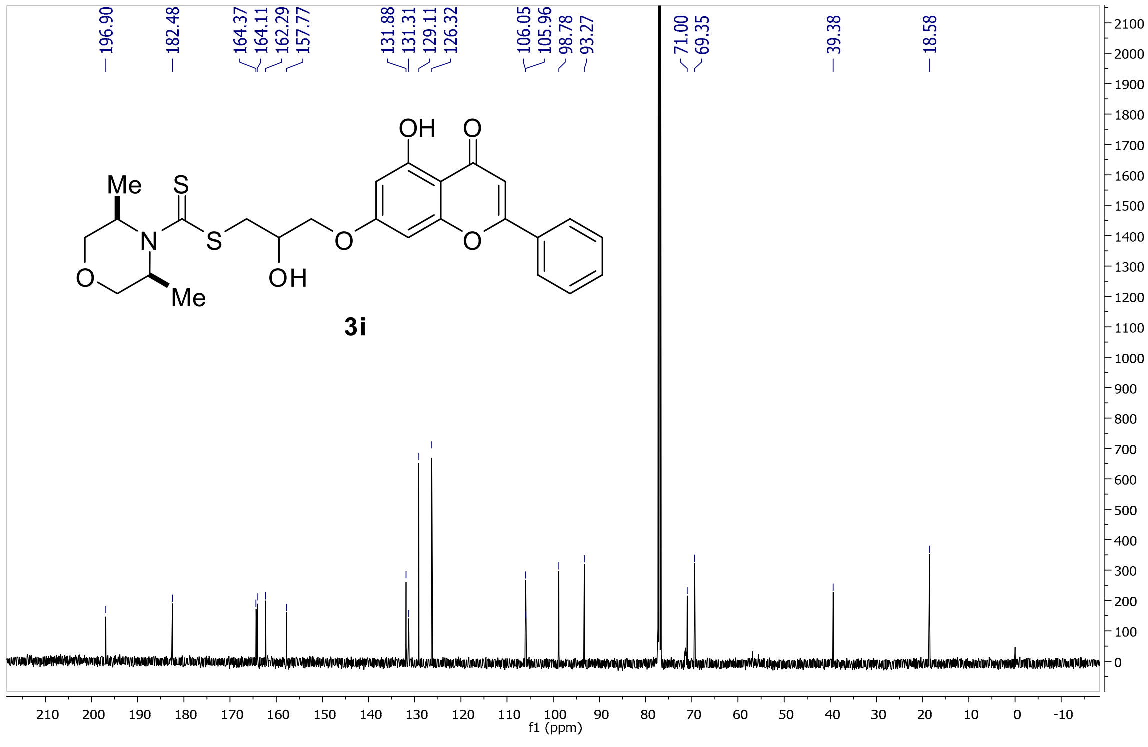

^{13}C NMR (125 MHz, $CDCl_3$) spectrum of compound **3i**

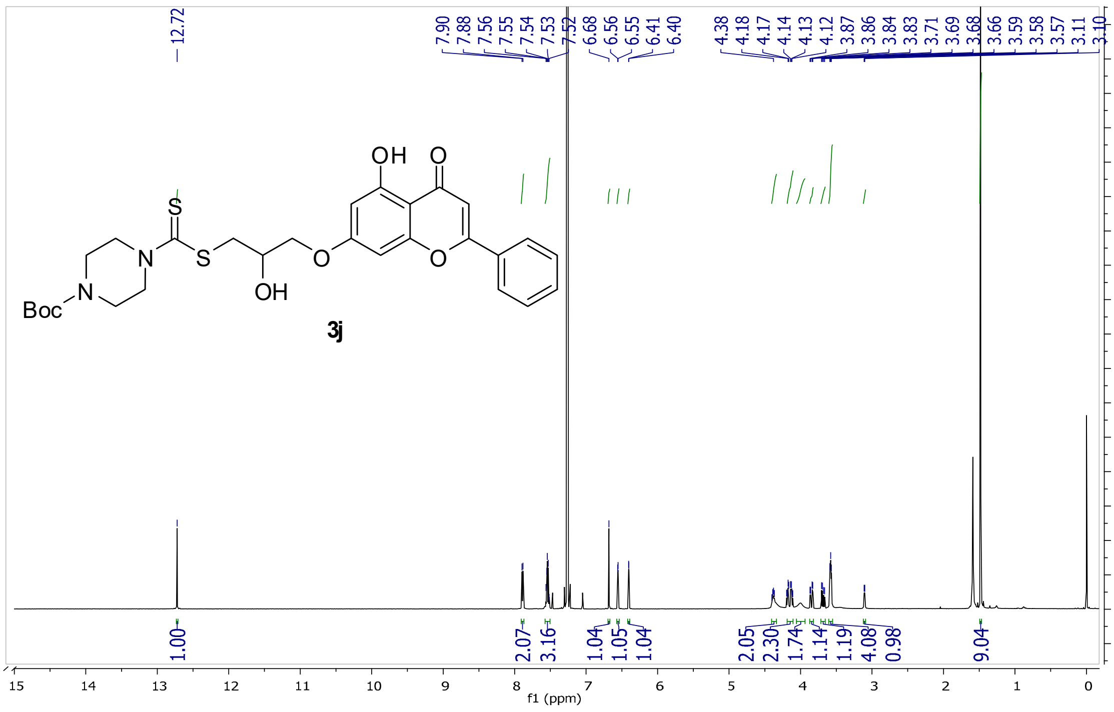

^{1}H NMR (500 MHz, $CDCl_3$) spectrum of compound **3j**

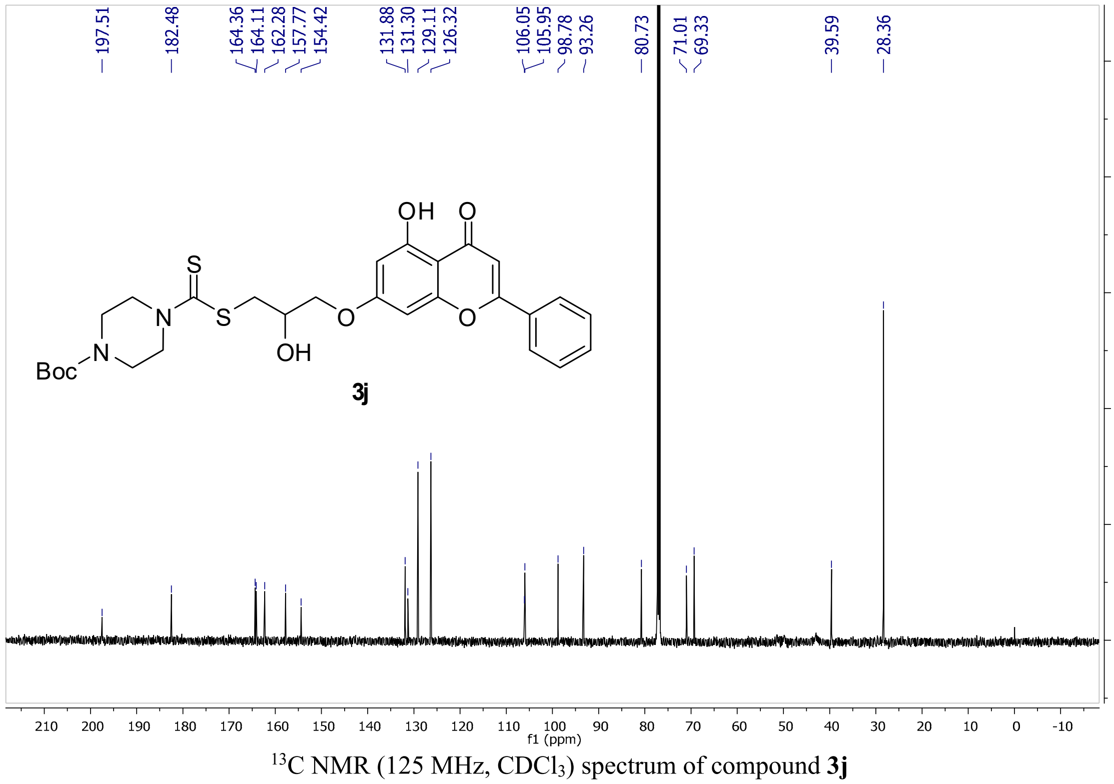

^{13}C NMR (125 MHz, $CDCl_3$) spectrum of compound **3j**

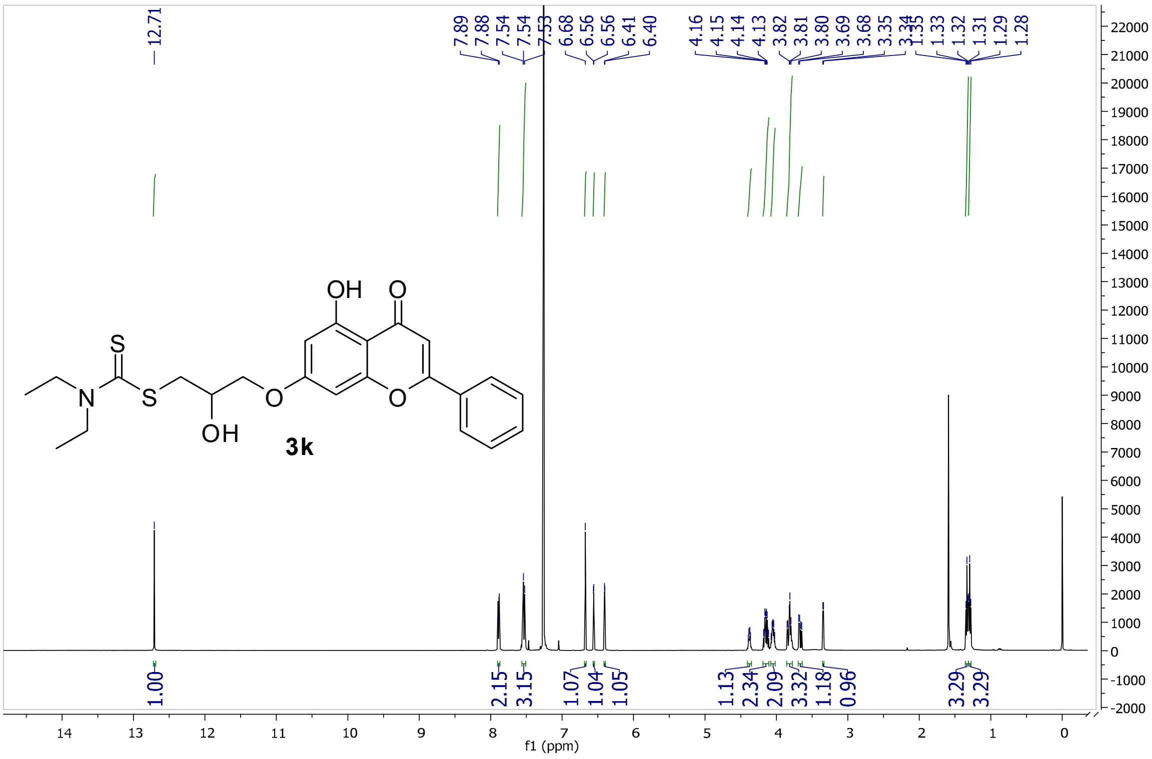

^{1}H NMR (500 MHz, $CDCl_3$) spectrum of compound **3k**

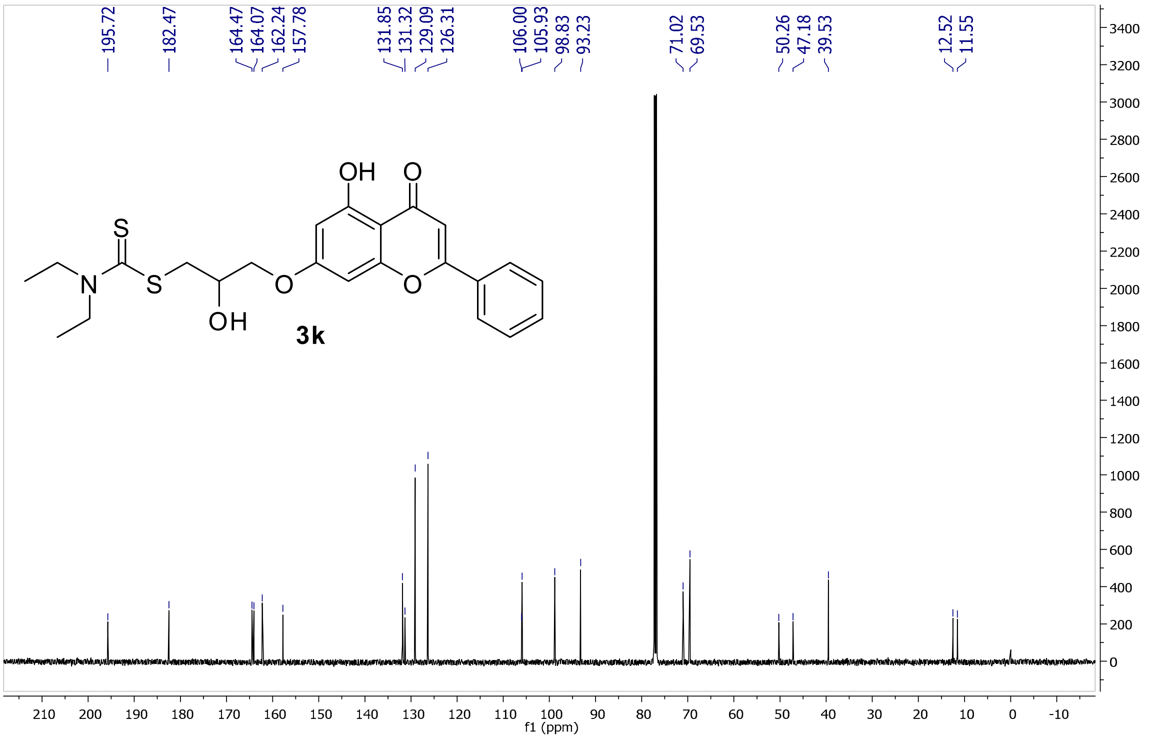

^{13}C NMR (125 MHz, $CDCl_3$) spectrum of compound **3k**
